# Genomic epidemiology and carbon metabolism of *Escherichia coli* serogroup O145 reflect contrasting phylogenies

**DOI:** 10.1371/journal.pone.0235066

**Published:** 2020-06-25

**Authors:** Rose M. Collis, Patrick J. Biggs, Anne C. Midwinter, A. Springer Browne, David A. Wilkinson, Hamid Irshad, Nigel P. French, Gale Brightwell, Adrian L. Cookson

**Affiliations:** 1 AgResearch Ltd, Hopkirk Research Institute, Massey University, Palmerston North, New Zealand; 2 Molecular Epidemiology and Veterinary Public Health Laboratory (^*m*^EpiLab), Infectious Disease Research Centre, School of Veterinary Science, Massey University, Palmerston North, New Zealand; 3 School of Fundamental Sciences, Massey University, Palmerston North, New Zealand; 4 New Zealand Food Safety Science and Research Centre, Massey University, Palmerston North, New Zealand; 5 Animal Health Programme, National Agricultural Research Centre, Islamabad, Pakistan; Defense Threat Reduction Agency, UNITED STATES

## Abstract

Shiga toxin-producing *Escherichia coli* (STEC) are a leading cause of foodborne outbreaks of human disease, but they reside harmlessly as an asymptomatic commensal in the ruminant gut. STEC serogroup O145 are difficult to isolate as routine diagnostic methods are unable to distinguish non-O157 serogroups due to their heterogeneous metabolic characteristics, resulting in under-reporting which is likely to conceal their true prevalence. In light of these deficiencies, the purpose of this study was a twofold approach to investigate enhanced STEC O145 diagnostic culture-based methods: firstly, to use a genomic epidemiology approach to understand the genetic diversity and population structure of serogroup O145 at both a local (New Zealand) (n = 47) and global scale (n = 75) and, secondly, to identify metabolic characteristics that will help the development of a differential media for this serogroup. Analysis of a subset of *E*. *coli* serogroup O145 strains demonstrated considerable diversity in carbon utilisation, which varied in association with *eae* subtype and sequence type. Several carbon substrates, such as D-serine and D-malic acid, were utilised by the majority of serogroup O145 strains, which, when coupled with current molecular and culture-based methods, could aid in the identification of presumptive *E*. *coli* serogroup O145 isolates. These carbon substrates warrant subsequent testing with additional serogroup O145 strains and non-O145 strains. Serogroup O145 strains displayed extensive genetic heterogeneity that was correlated with sequence type and *eae* subtype, suggesting these genetic markers are good indicators for distinct *E*. *coli* phylogenetic lineages. Pangenome analysis identified a core of 3,036 genes and an open pangenome of >14,000 genes, which is consistent with the identification of distinct phylogenetic lineages. Overall, this study highlighted the phenotypic and genotypic heterogeneity within *E*. *coli* serogroup O145, suggesting that the development of a differential media targeting this serogroup will be challenging.

## Introduction

Shiga toxin-producing *Escherichia coli* (STEC) are zoonotic pathogens residing harmlessly in the gut of bovine reservoirs, but capable of causing human disease with a broad range of symptoms; from diarrhoea to life-threatening haemolytic uraemic syndrome (HUS) [1, 2]. STEC can be shed in large numbers in faeces excreted by ruminants [3, 4], particularly calves [5], and are an important source of both foodborne and environmentally acquired STEC infections through direct contact with faeces or faecally-contaminated environments. Most human infections are associated with sporadic outbreaks where risk factors include contact with cattle, animal manure, recreational waters [6] or consumption of contaminated food [7]. STEC have been identified as the causative pathogenic agent in disease outbreaks associated with a wide variety of contaminated food products such as romaine lettuce [8], ice-cream [9], and hamburger patties [10]. In an attempt to manage food-related risk, seven serogroups (O26, O45, O103, O111, O121, O145 and O157) collectively described as the ‘Top 7’ have been declared adulterants of ground beef in the United States of America (USA) [11, 12] impacting food safety regulations and international trade. A cross-sectional study investigating the prevalence of STEC in young calves (2–21 days of age) throughout New Zealand (NZ) identified STEC O145 as the most prevalent serogroup (43%) at the dairy farm level compared with the other ‘Top 7’ serogroups [13]. These prevalence data indicate that, as a zoonotic pathogen, *E*. *coli* serogroup O145 represents both a risk to public health and a regulatory issue for NZ’s meat export industry.

STEC express Shiga toxins encoded by the *stx1* and *stx2* genes within lambdoid bacteriophage [14] maintained in a lysogenic state [15]. Stx toxin production is a component of STEC pathogenesis in humans that occurs during bacterial adhesion and intestinal colonisation, leading to impaired intestinal epithelial cell barrier function and diarrhoea [16]. Systemic dissemination of Stx toxin through the cardiovascular system may also lead to HUS and other sequelae [16]. Other important virulence factors for STEC pathogenicity include enterohaemolysin, a plasmid-associated pore-forming RTX toxin encoded by the *ehxA* gene [17, 18] and an outer membrane adhesin, intimin, encoded by the *eae* gene located within the Locus of Enterocyte Effacement (LEE) pathogenicity island [19]. Intimin and other LEE-encoded type III secretion system components and effector proteins mediate the formation of attaching and effacing lesions [19], which are actin pedestals characterised by microvilli effacement and bacterial attachment to the intestinal epithelial cells [19]. The C-terminal end of intimin has a highly variable amino acid sequence thought to be associated with contrasting host tissue tropisms [20, 21] to the extent that the *eae* gene has been differentiated into at least 28 different subtypes [22]. Some STEC serotypes are characterised by a single *eae* subtype such as O157:H7 (γ), O26:H11 (β), O103:H2 (ε), O111:H8 (θ) and O145:H28 (γ) [23], however, multiple *eae* subtypes may be associated with other serogroups [19, 24]. The LEE pathogenicity island is inserted in the *E*. *coli* genome near tRNA genes such as *selC*, *pheV* and *pheU* [19] and is found in enteropathogenic *E*. *coli* (EPEC), that lack *stx* genes, in addition to the STEC pathotype [25]. Importantly, many STEC do not possess the *eae* and *ehxA* molecular markers; for example, a large foodborne outbreak was caused by a hybrid STEC/Enteroaggregative *E*. *coli* O104:H4 strain which was *stx2*-positive and negative for both *eae* and *ehxA* [26, 27], suggesting that all STEC should be treated as pathogenic, regardless of specific O-serogroups [28]. These distinct diarrhoeagenic *E*. *coli* pathotypes such as STEC and EPEC are often identified according to the presence or absence of specific virulence factors, such as the *stx* and *eae* genes, but display significant genetic heterogeneity and readily acquire new genetic material via horizontal gene transfer (HGT) [25].

Current culture-based detection methods for non-O157 STEC do not provide sufficient discrimination between serogroups due to the lack of differential characteristics between non-O157 STEC serogroups in comparison to non-pathogenic *E*. *coli* [29, 30]. A variety of selective media currently available have been developed for the detection and isolation of STEC utilising carbohydrate fermentation patterns to detect specific serogroups based on colony colour [9, 30]. Such media containing carbon substrates have recently been proposed to differentiate the serogroups O26, O103, O111, O145 and O157 [31], in conjunction with other previously developed methods [32]. However, the efficacy of such media utilising these substrates has not been fully validated [31], and this is likely to be a key factor associated with highly variable isolation rates of non-O157 STEC serogroups between studies [29, 33–35] and their probable under-reporting [36]. Despite the extra efforts required with culture-based techniques for the isolation of non-O157 serogroups such as O145, in comparison to rapid molecular methods [37, 38], the identification and isolation of individual bacterial strains provides subsequent opportunities for further epidemiological and clinical analysis [30, 35, 38]. Other studies have analysed data from large panels of serogroup-specific STEC strains [39], such as environmental and clinical isolates [40], but these isolates are often associated with a distinct geographical area [40].

Whole genome sequencing (WGS) provides the ability for high-resolution genetic typing analysis that can be used in epidemiological investigations whilst simultaneously providing information on an isolate’s gene content. Previous studies to provide STEC serogroup phylogenies have been limited through the analysis of WGS data from a limited number of serogroup-specific strains [41–43], or from datasets biased towards human isolates [44], causing wide-ranging serogroup-specific diversity to be over looked.

Therefore, the purpose of this study was firstly, to take a broad-ranging approach to understand the genetic diversity and population structure of serogroup O145 at both a local (NZ) and a global scale using genomic epidemiology methods and, secondly, to identify characteristic metabolic traits associated with serogroup O145 which could prove beneficial in the development of culture-dependent tests for this serogroup.

## Materials and methods

### *E*. *coli* serogroup O145 strains

In this study, 53 *E*. *coli* serogroup O145 strains (S1 Table) were whole genome sequenced from NZ (n = 47), Norway (n = 4), Australia (n = 1) (provided by Roy Robbins-Browne, University of Melbourne) and USA (n = 1). These were isolated from bovine (n = 36), environmental (n = 6) and human clinical sources (n = 11). The serogroup of these O145 strains (S1 Table) was confirmed using an O145 serogroup-specific PCR, with the primers (5’-GCGGGTGTTGCCCGTTCTGT-3’) and (5’-ACGGCATTCCGCTGCGAGTT-3’) [29] and subsequently with analysis of WGS data. Whole genome sequence data from an additional 69 overseas isolates (human clinical cases: n = 36; bovine: n = 12; food: n = 3; ground beef: n = 1; intact beef: n = 1; ground pork: n = 2; intact pork: n = 3; swine: n = 3; wolf: n = 2; and unknown: n = 6) were included in the comparative genome analysis (S2 Table) to provide a global context. Most of the 122 isolates analysed in the global study were from New Zealand (n = 47) and the USA (n = 47) with the remainder from the UK (n = 15), Norway (n = 4), Canada (n = 2) and one each from Australia, Denmark, Germany, Italy and Uruguay. Two isolates were of unknown geographic origin.

### DNA extraction, library preparation and whole genome sequencing

Previously described DNA extraction and library preparation methods [13] were used to prepare the *E*. *coli* serogroup O145 isolates for WGS. Aliquots of the first four library preparations and an aliquot of the pooled libraries underwent a quality control check (Bioanalyzer 2100 [Agilent Genomics, Santa Clara, CA, USA]) at New Zealand Genomics Limited (NZGL, Massey Genome Service, Massey University, Palmerston North, New Zealand). WGS was performed by NZGL (University of Otago, Dunedin, New Zealand) using an Illumina HiSeq paired-end v4 platform (2 x 125 bp).

### Genome quality control, assembly and annotation

The raw sequencing reads were evaluated using quality control software (QCtool) [45]. The sequences were *de novo* assembled using SPAdes v3.9.1 [46].The quality assessment tool QUAST was used to assess and compare the quality of the genome assemblies [47], which were annotated using Prokka (v1.12-beta) [48]. Genome assembly statistics are displayed in the supplementary information (S1 Fig).

### Downloading publicly available serogroup O145 raw sequence data

Serogroup O145 strains were identified from NCBI [49], EnteroBase [50] and published papers (S2 Table). Only whole genome sequences in which the raw read sequence data was available were further analysed using the same analysis pipeline (namely quality assessment, assembly and genome analysis). Publicly available whole genome sequences were excluded from the analysis if any discrepancies indicative of potential contamination such as genome size (<4 Mb or >6Mb) or GC content (<48% or >51%) were identified during the quality assessment, or if an over-representation of unassigned/ambiguous nucleotides (Ns) in the reads was identified using FastQC, or if the identity of the *wzx* and *wzy* genes of the O-antigen biosynthesis gene cluster could not be confirmed as homologous to those from serogroup O145.

### Genetic characterisation of *E*. *coli* serogroup O145 strains

Assembled genomes were batch uploaded to the Center for Genomic Epidemiology (CGE) server [51] for identification of serotype (O and H antigens; threshold of 85% identity (ID) and a minimum gene fractional length of 60%) [52], species [53], *E*. *coli* associated virulence factors (n = 76; threshold of 90% ID and a minimum gene fractional length of 60%) [54], plasmids (threshold of 95% ID and a minimum gene fractional length of 60%) [55], antibiotic resistance genes (threshold of 90% ID and a minimum gene fractional length of 60%) [56] and multi-locus sequence typing (MLST) [57]. The *stx* variants were determined by VirulenceFinder, and the *eae* subtype was determined by identifying the best nucleotide match using BLASTN [58]. The EPEC-associated bundle forming pilus subunit *bfpA* [59] was detected using Geneious v8.1 [60]. *In silico* analysis of ribosomal multi-locus sequence types (rMLST) [61] was generated from single nucleotide polymorphisms identified in 51 genes encoding the ribosome protein subunits (*rps*, *rpm* and *rpl*). The *in silico* rMLST analysis was visualised using neighbour-joining methods in SplitsTree v4.14.4 [62] and edited using the Interactive Tree of Life (iTOL) webserver [63].

The presence or absence of 37 virulence genes identified using VirulenceFinder [54], which differed between strains, were used to make a Neighbour-joining tree using the Jaccard index and converted to the Newick file format using R 3.6.0 [64] and the packages ‘vegan’ [65] and ‘ctc’ [66]. The tree was edited using the iTOL webserver [63] and isolate metadata was included for *eae* subtype, sequence type (ST) and isolation source.

The LEE pathogenicity island integration sites were identified using either the location of LEE-encoded genes including a prophage integrase adjacent to a potential tRNA (*selC*, *pheU* or *pheV*) integration site, or contigs were assembled to a reference genome and the likely tRNA integration predicted based on the mapped contigs and gene synteny using Geneious v8.1 [60]. The reference genomes used were STEC O145:H28 RM13514 (NZ_CP006027) or STEC O145:H28 RM12761 (NZ_CP007133) where the LEE is integrated at tRNA *selC* [43], STEC O26:H11 11368 (AP010953) [67] where the LEE is integrated at tRNA *pheU* or STEC O103:H2 12009 (AP010958) where the LEE is integrated at tRNA *pheV* [67].

### Comparative genomics

Single nucleotide polymorphisms (SNPs) were identified in the paired-end sequencing reads using Snippy v3.0 [68] and STEC O145:H28 RM12761 (NZ_CP007133), associated with a foodborne STEC outbreak in Belgium, was used as the reference genome [43]. This isolate has three contigs (a chromosome and two large plasmids) and has a virulence profile similar to several of the O145 isolates in this study (*stx*-positive, *eae* subtype γ). At the time of this study, there were no publicly available genome sequences from STEC O145 isolated in NZ. Randomised Axelerated Maximum Likelihood (RAxML) Next-Generation [69] maximum-likelihood trees were generated of the core SNP alignment using a general time-reversible model and random seed to perform 20 tree searches using ten random and ten parsimony-based starting trees. The best-scoring maximum-likelihood tree was viewed in iTOL [63]. Roary [70] was used to identify the pangenome and the core and accessory genes in the *E*. *coli* serogroup O145 strains.

### Accession numbers

The accession numbers for *E*. *coli* serogroup O145 strains whole genome sequenced in this study are listed in S3 Table and are deposited with NCBI under the BioProject number PRJNA435641.

### Biolog phenotypic microarray assays

The Omnilog phenotypic microarray system (Biolog Inc, Hayward, California, USA) was used to investigate the metabolic characteristics of serogroup O145 strains. Serogroup O145 strains to be examined were selected using random sampling, stratified by the variables: *eae* subtype, ST, the geographic origin of isolation and a *stx*-positive or *stx*-negative genotype (Table 1). The plates were prepared as previously described [31], except the colonies were re-suspended at a light transmittance of 42%. Half of the isolates (n = 14) were tested in replicate (analysed on separate days) and two in duplicate (analysed on the same day) for the PM1 MicroPlates^TM^, and four biological replicates were tested for the PM2A MicroPlates^TM^. The raw Omnilog data was analysed using R 3.3.1 [64] and the packages ‘opm’ [71] and ‘gplots’ [72]. To compare carbon substrate utilisation between the strains, the end-point values per serogroup O145 strain (n = 28) for each carbon substrate on the phenotypic microarray plates (n = 95) was recorded and used to produce a cluster dendrogram using hierarchical clustering, with height indicating the distance between pairs.

**Table 1:** Serogroup O145 strains analysed using the Omnilog phenotypic microarray system.

Strain	MicroPlates™	Serotype^a^	Source	Origin	Sequence type	Virulence profile	*eae* subtype
Trh7	PM1, PM2A^b^	O145:H40	Human	Norway	ST-10	*eae*	β
TW07865	PM1^b^, PM2A	O145:H28	Human	Germany	ST-137	*stx2*, *eae*, *ehxA*	γ
AGR718	PM1^c^	O145:H46	Bovine	Manawatu, New Zealand	ST-137	*eae*, *ehxA*	γ
ERL020412	PM1^c^	O145:H28	Human	New Zealand	ST-137	*eae*, *ehxA*	γ
16ER0267A	PM1^b^, PM2A^b^	O145:H2	Human	Auckland, New Zealand	ST-17	*stx1*, *eae*, *ehxA*	ε
16ER0517A	PM1^b^, PM2A	O145:H2	Human	Auckland, New Zealand	ST-17	*stx1*, *eae*, *ehxA*	ε
116B	PM1, PM2A	O145:H2	Bovine	Taranaki, New Zealand	ST-17	*eae*, *ehxA*	ε
188B	PM1	O145:H2	Bovine	Taranaki, New Zealand	ST-17	*eae*, *ehxA*	ε
267P	PM1^b^, PM2A	O145:H2	Bovine	Taranaki, New Zealand	ST-17	*eae*, *ehxA*	ε
54B	PM1^b^	O145:H2	Bovine	Taranaki, New Zealand	ST-17	*eae*, *ehxA*	ε
13ER3103A	PM1, PM2A	O145:H28	Human	Auckland, New Zealand	ST-32	*stx2*, *eae*, *ehxA*	γ
VC1281m	PM1^b^, PM2A	O145:H28	Bovine	Canterbury, New Zealand	ST-32	*eae*, *ehxA*	γ
VC847m	PM1	O145:H28	Bovine	Manawatu-Wellington, New Zealand	ST-32	*eae*, *ehxA*	γ
13ER4824	PM1	O145:H28	Bovine	New Zealand	ST-32	*stx2*, *eae*, *ehxA*	γ
13ER5640	PM1, PM2A	O145:H28	Bovine	New Zealand	ST-32	*stx2*, *eae*, *ehxA*	γ
14ER2392	PM1, PM2A	O145:H28	Bovine	New Zealand	ST-32	*stx2*, *eae*, *ehxA*	γ
H12ESR01231	PM1^b^, PM2A	O145:H28	Bovine	New Zealand	ST-32	*eae*, *ehxA*	γ
H12ESR01387	PM1, PM2A	O145:H28	Bovine	New Zealand	ST-32	*stx2*, *eae*, *ehxA*	γ
H12ESR03525	PM1, PM2A	O145:H28	Bovine	New Zealand	ST-32	*stx2*, *eae*, *ehxA*	γ
VC308m	PM1^b^	O145:H28	Bovine	Northland, New Zealand	ST-32	*eae*, *ehxA*	γ
Trh30	PM1, PM2A	O145:H28	Human	Norway	ST-32	*eae*, *ehxA*	γ
VC1413m	PM1^b^, PM2A^b^	O145:H28	Bovine	Southland, New Zealand	ST-32	*stx2*, *eae*, *ehxA*	γ
VC1506m	PM1, PM2A	O145:H28	Bovine	Southland, New Zealand	ST-32	*eae*, *ehxA*	γ
F5J	PM1	O145:H2	Environmental	Waikato, New Zealand	ST-32	*eae*, *ehxA*	γ
P2B1	PM1^b^, PM2A	O145:H28	Environmental	Waikato, New Zealand	ST-32	*eae*, *ehxA*	γ
Trh42	PM1^b^, PM2A	O145:H34	Human	Norway	ST-35	*eae*	ι
13ER6723A	PM1^b^, PM2A	O145:H34	Human	Auckland, New Zealand	ST-722	*stx2*, *eae*	ι
R249-1	PM1^b^, PM2A^b^	O145:H34	Human	Australia	ST-722	*eae*	ι

a: O antigen: H antigen

b: MicroPlates™ were completed in replicate (analysed on separate days)

c: MicroPlates™ were completed in duplicate (analysed on the same day)

## Results and discussion

### Population structure and genome composition of *E*. *coli* serogroup O145 strains

**Fig 1:**
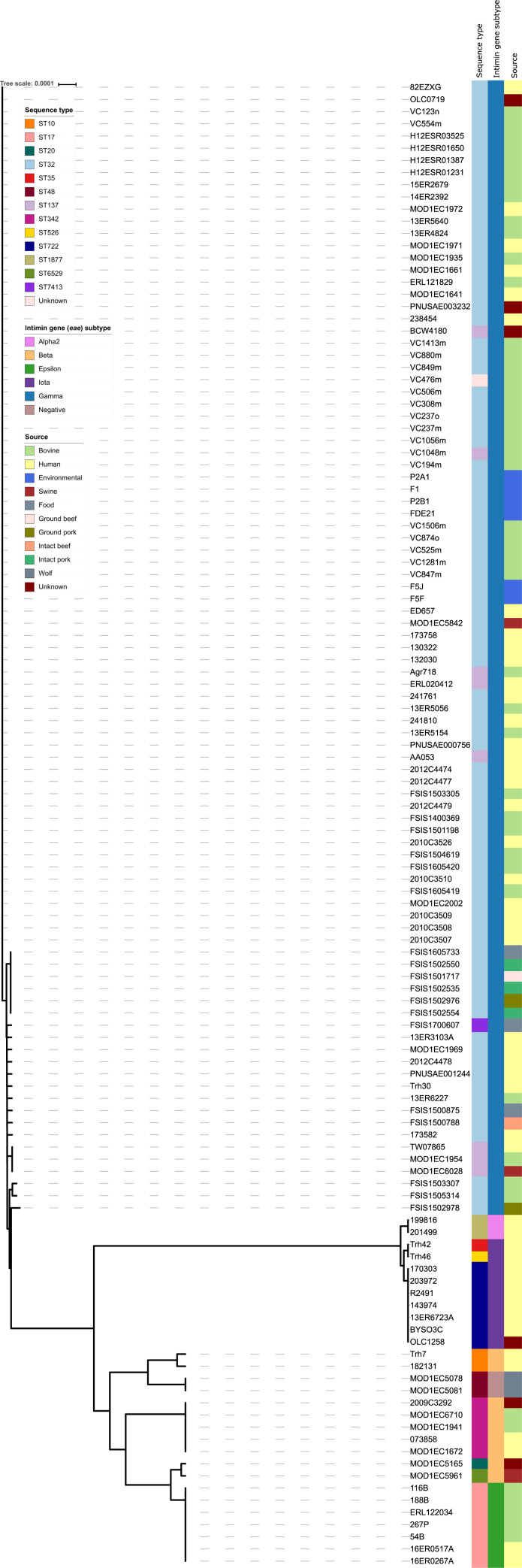
Neighbour-joining phylogeny constructed using *in silico* ribosomal multi-locus sequence typing. A Neighbour-joining tree of ribosomal multi-locus sequence types (rMLST) generated from single nucleotide polymorphisms identified in 51 genes encoding the ribosome protein subunits (*rps*, *rpm* and *rpl*). The *in silico* rMLST analysis was visualised using neighbour-joining methods in SplitsTree and edited using the iTOL (Interactive Tree of Life) webserver. Isolate metadata is included for sequence type, *eae* subtype and isolation source, as indicated by the colour keys.

A total of 122 *E*. *coli* serogroup O145 strains were analysed, 53 sequenced in this study (S1 Table) and 69 publicly available genome sequences (S2 Table). Using the Achtman MLST system [73], a total of 14 distinct STs were identified for the 122 isolates (Fig 1). The predominant type was ST32 (83 of 122, 68.0%), followed by ST137 (8 of 122, 6.6%), ST17 and ST722 (7 of 122, 5.73% each), ST342 (5 of 122, 4.1%), ST10, ST48 and ST1877 (2 of 122, 1.64% each) and ST20, ST35, ST526, ST6529, ST7413 and ST unknown (1 of 122, 0.82% each). ST32 was also found to be predominant (230 of 239, 96.2%) in a study of O145:H28 strains [44]. Each ST was also associated with a specific *eae* subtype, as highlighted in the neighbour-joining tree generated from SNPs identified in 51 genes encoding the ribosome protein subunits (Fig 1).

**Fig 2:**
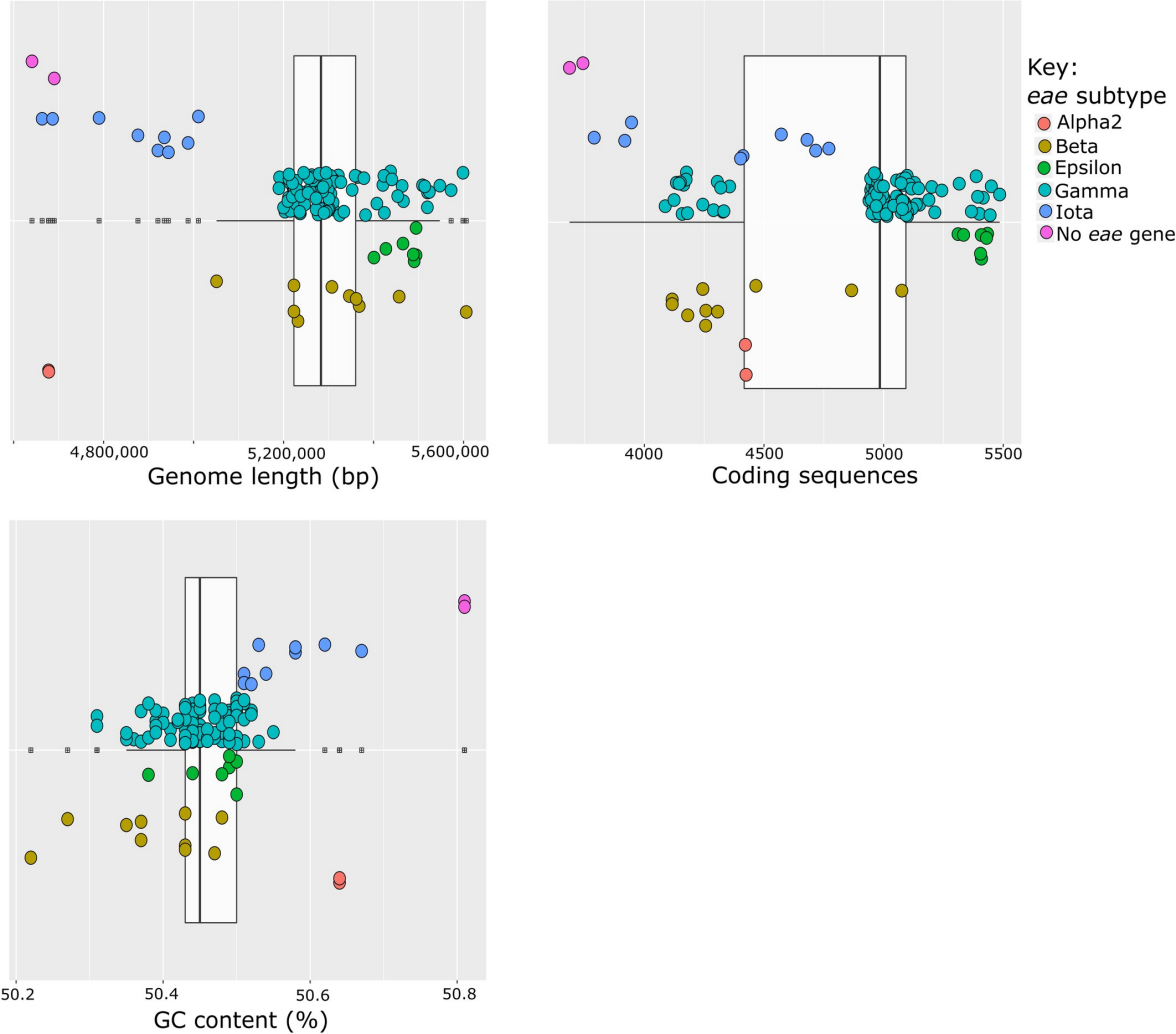
Box and whisker plots indicating the genome composition of *E*. *coli* serogroup O145 strains (n = 122). The box and whisker plots indicate the genome length (bp), number of coding sequences and GC content (%) for the serogroup O145 strains (n = 122). The box spans the interquartile range, with the lower and upper quartiles indicated by the ends of the box (from left to right) and the median by the vertical line inside the box. Each data point is shown on the plots and has been grouped according *eae* subtype in the y axes, as indicated by the figure key.

A summary of the genome composition for the *E*. *coli* serogroup O145 strains (n = 122) is shown in Fig 2, indicating genome length (bp), coding sequence counts (CDS) and GC content (%). For all three parameters, clustering occurred according to *eae* subtype. The shortest genome lengths were associated with *eae*-negative, *eae* subtype ι and α2 strains (4,640,737–5,010,707 bp). Similarly, the *eae*-negative and three *eae* subtype ι strains also had the lowest CDS counts (3,687–3,946). The CDS counts for the remaining *eae* subtype ι strains (n = 6), *eae* subtype α2, β, ε and λ strains ranged from 4,087 to 5,485. The *eae* subtype ε strains had both the longest genome length (5,400,785–5,494,427 bp) and highest CDS counts (5,311–5,434). The *eae*-negative strains had a slightly higher GC content (50.81%), however, the GC content of all serogroup O145 strains was relatively similar (50.22–50.81%).

### Genetic characterisation of virulence factors and antimicrobial resistance

**Fig 3:**
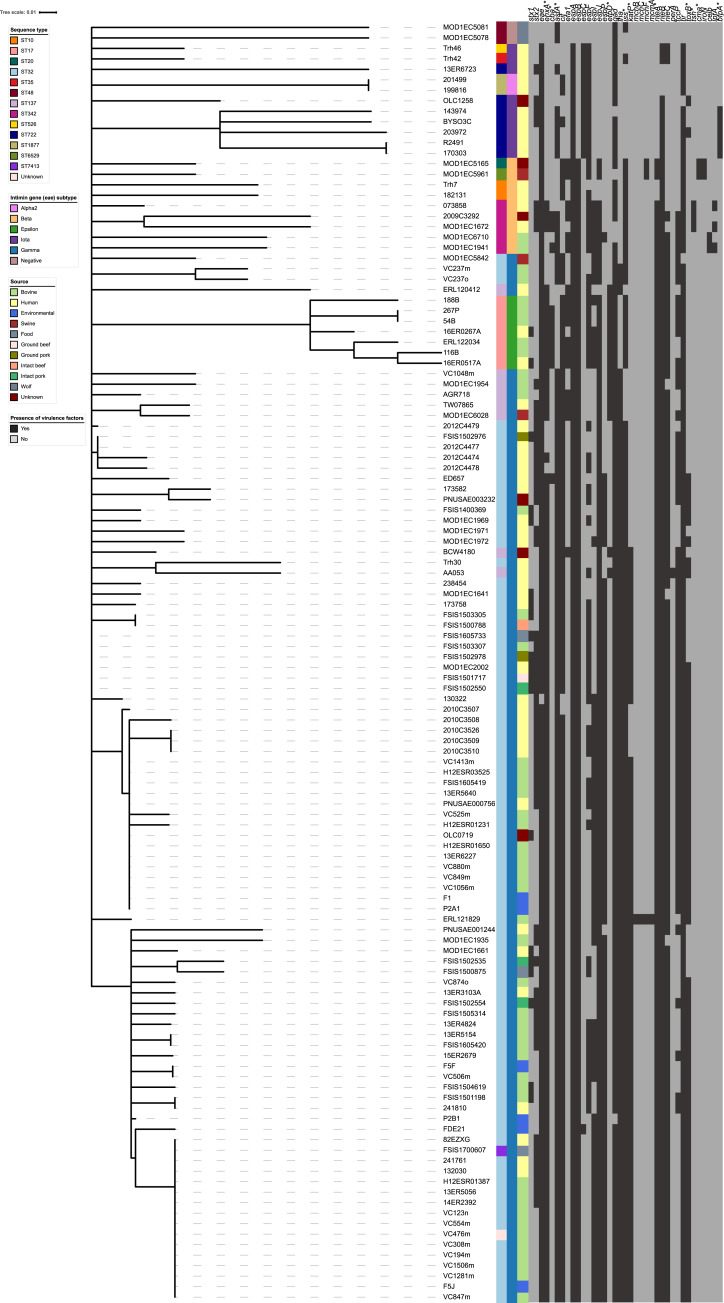
Neighbour-joining tree constructed using the presence or absence data from 37 virulence genes identified by the center for genomic epidemiology VirulenceFinder webserver.

The virulence profiles of the serogroup O145 isolates (n = 122) (S4 Table) broadly cluster according to both *eae* subtype and ST (Fig 3). The *eae* subtype γ (n = 93) strains cluster together, with some variation according to ST and at the strain level. WGS data analysis of the *eae* subtype γ strains indicates the presence of between 13 and 22 of the 37 virulence factors, as shown in Fig 3. Notably, the plasmid-associated virulence factor *etpD* was present in the eight ST137 strains and absent in the remaining ST32 and ST7413 (n = 85) *eae* subtype γ strains.

The presence or absence of 37 virulence genes identified using VirulenceFinder, which differed between strains, were used to make a Neighbour-joining tree using the Jaccard index and converted to Newick format using R 3.6.0 and the packages ‘vegan’ and ‘ctc’, respectively. The tree was edited using the iTOL webserver and isolate metadata was included for *eae* subtype, sequence type (ST) and isolation source.

The *eae* subtype α2 (n = 2), ι (n = 9), *eae*-negative (n = 2), β (n = 9) and ε (n = 7) strains broadly form separate clusters, with similarity within each cluster correlating with ST. The *eae* subtype ε strains (n = 7, ST17) carry between 17 and 19 virulence factors, and the *eae* subtype β strains carry between 8 and 19 virulence factors. In comparison to other serogroup O145 strains, the α2, ι, β (ST10) and *eae*-negative strains carry fewer virulence factors; *eae* subtype α2, ι, β (ST10) and *eae*-negative strains carry 7, 8 to 11, 8 and 9, and 2 and 3 of the 37 virulence factors listed in Fig 3, respectively. The low number of virulence factors carried by the *eae*-negative and *eae* subtype α2, ι and β (ST10) strains may be partially due to the absence of plasmid-acquired virulence factors, such as *etpD*, *ehxA* and *katP* (Fig 3). Five strains (ST722, *eae* subtype ι) are defined as typical EPEC due to the presence of the LEE pathogenicity island and the bundle forming pilus biosynthesis operon [74].

Of the 122 isolates, 65 were *stx*-positive (53.3%) including *stx* variants *stx1a* (12 of 65, 18.5%), *stx2a* (35 of 65, 53.8%), *stx2c* (2 of 65, 3.1%), *stx2d* (4 of 65, 6.2%) and *stx2f* (4 of 65, 6.2%). Eight isolates were both *stx1* and *stx2* positive (*stx1a*, *stx2a*, 1 of 65, 1.5%; *stx1a*, *stx2d*, 7 of 65, 10.7%). The remaining 57 isolates were *stx*-negative. Similar *stx* variants were detected in an analysis of 239 O145:H28 strains [44]; however, *stx2f* was not detected and was only detected in four O145:H34 strains in this study. It has been suggested that different *stx* variants may have varying levels of virulence, for example, *stx2a* has been associated with an increased risk of developing HUS [75, 76], in comparison *stx1* variants were associated with a lower risk [75]. STEC possessing the *stx2f* toxin, first described in pigeons [77], have been described as an emerging pathogen [78]. Preliminary epidemiological data suggested infections caused by *stx2f-*positive STEC were associated with mild clinical disease [79], however, one case of HUS caused by a STEC strain possessing *stx2f*, and *eae* positive and *ehxA* negative, has been reported [80]. Three of the four *stx2f* isolates were from human clinical cases, with the source of the remaining isolate being unknown.

Plasmids were detected in 113 out of 122 strains (S5 Table), with distinct nucleotide matches (≥95% identity and ≥60% coverage) of plasmid incompatibility factors indicative of separate plasmids. A single plasmid was identified in 89 O145 strains, two plasmids in 18, three plasmids in five and a single O145 strain was identified with four plasmids. The most commonly detected plasmid incompatibility factor was IncFIB (AP001918) which was detected in 96.5% (109 out of 113) of the strains. Interestingly, the IncFIB and IncB/O/K/Z_3 plasmids were found to be highly conserved within a population of 239 O145:H28 strains [44]. The ubiquity of the plasmid incompatibility factor IncFIB detected in this study may suggest that this plasmid is conserved within the O145 serogroup. Plasmid negative strains (n = 9), belonged to *eae* subtype α2 (n = 2), ι (n = 3), and γ (n = 4).

The LEE pathogenicity island integration sites were identified in 73 out of 120 serogroup O145 strains and are listed in S6 Table. A tRNA *phe*V integration site was identified for *eae* subtype ε (n = 7), β (n = 2) and γ (n = 2) strains, *pheU* for the *eae* subtype β (n = 1) strain and *selC* for the *eae* subtype α2 (n = 2), β (n = 1), ι (n = 7), and γ (n = 49) strains. Although the *selC* LEE integration site for *eae* subtype ι strains has not previously been identified, the precise LEE integration site could not be determined for one *eae* subtype ι strain Trh42 but was located near tRNA *leu*. This potential LEE insertion site was also observed for the *eae* subtype β strain 73858. The LEE insertion site could not be determined in 47 strains, likely due to incomplete genome assemblies as a result of using short-read sequencing. The common *stx*-bacteriophage insertion sites for serogroup O145 [81] were analysed to identify whether these sites were occupied or available in *stx*-negative strains. Although some sites were vacant in the majority of *stx*-negative strains, not all insertion sites could be detected. This was possibly due to the genes surrounding these sites being unannotated; the sites being occupied and the insertion site therefore disrupted; or the genome assembly being incomplete. The detection of *stx*-bacteriophage insertion sites in serogroup O145 isolates is problematic due to multiple potential insertion sites, variations in prophage structure and variation between integration sites, including between phage which encode the same Stx subtype [44]. In addition, “Stx_2_-like” prophage, which appear to be defective as a result of nonsense mutations in the *stx*_*2*_A subunit or absent *stx*_*2*_A and *stx*_*2*_B genes, have been detected in serogroup O145 strains [42], further complicating the detection of *stx*-bacteriophage insertion sites in this serogroup.

The serogroup O145 genome sequences (n = 122) were examined for antibiotic resistance genes using ResFinder [56]. Twenty-three of 122 strains (18.9%) carried one or more resistance genes with resistance to up to a maximum of five classes of antibiotics being detected (Table 2). These strains were from a variety of sources, belonged to multiple STs and *eae* subtypes and were isolated in the USA (n = 17), UK (n = 2), NZ (n = 1), Germany (n = 1) and Canada (n = 1) with the geographic isolation of one strain being unknown. Notably, all 23 of the resistant strains carried genes conferring aminoglycoside resistance. The variation in the carriage of antibiotic resistance genes in the serogroup O145 genomes may be a result of varying selective pressures that may impact the development and transmission of resistance, such as antimicrobial use in different geographical regions. For example, only one out of 35 serogroup O145 strains isolated from bovine sources in NZ carried an antibiotic resistance gene; which may be reflective of the low antimicrobial use in the dairy industry in NZ [82]. As a result of this variability, it is unlikely that antimicrobial resistance is a property that could be utilised in the development of a media for the differentiation of serogroup O145.

**Table 2:** Detection of genes conferring resistance to certain classes of antibiotics.

Strain	Origin	Source	Sequence type	Antibiotic classes				
				Aminoglycoside	Beta-lactam	Phenicol	Sulphonamide	Tetracycline
182131	UK	Human	10	+	+	-	-	-
MOD1EC5165	Unknown	Unknown	20	+	+	-	+	-
OLC0719	Canada	Unknown	32	+	-	-	+	+
VC874o	NZ	Bovine	32	+	-	-	+	+
173758	UK	Human	32	+	-	-	+	-
FSIS1605420	USA	Bovine	32	+	-	+	+	+
FSIS1605419	USA	Bovine	32	+	-	+	+	+
MOD1EC1935	USA	Bovine	32	+	-	-	+	+
2010C3507	USA	Human	32	+	-	+	+	+
2010C3508	USA	Human	32	+	-	+	+	+
2010C3509	USA	Human	32	+	-	+	+	+
2010C3510	USA	Human	32	+	-	+	+	+
2010C3526	USA	Human	32	+	-	+	+	+
MOD1EC1971	USA	Human	32	+	-	+	+	+
MOD1EC1972	USA	Human	32	+	-	+	+	+
MOD1EC5842	USA	Swine	32	+	-	-	+	+
MOD1EC1954	Germany	Bovine	137	+	+	+	+	+
TW07865	USA	Human	137	+	+	+	+	+
MOD1EC6028	USA	Swine	137	+	+	+	+	+
MOD1EC6710	USA	Bovine	342	+	+	-	+	+
MOD1EC1941	USA	Bovine	342	+	+	-	+	+
2009C3292	USA	Unknown	342	+	-	-	-	-
MOD1EC5961	USA	Swine	6529	+	+	-	+	+

Comparative genomics of the 122 serogroup O145 strains from diverse geographical regions and distinct hosts/sources highlighted the genetic heterogeneity within this serogroup. The strains analysed belonged to 14 different STs, carried between 2 and 22 *E*. *coli* associated virulence factors and 18.9% (23 out of 122) carried genes known to confer antibiotic resistance (Figs 1 and 3, and Table 2). Genome analysis indicated strains of the same *eae* subtype had a similar genome size and number of CDS counts (Fig 2), consistent with other ‘Top 7’ serogroups [42–44, 67, 83]. However, the *eae*-negative and *eae* subtype α2 and ι strains had smaller genomes (Fig 2) compared to other serogroup O145 strains. The WGS data from α2 and ι strains in this study are consistent with the genome parameters of two further atypical (bundle forming pilus negative) EPEC (aEPEC) O145:H34 isolates [84] with identical STs and *eae* subtypes (α2 and ι) recently analysed (S1 and S2 Tables).

### Core and pangenome analysis

**Fig 4:**
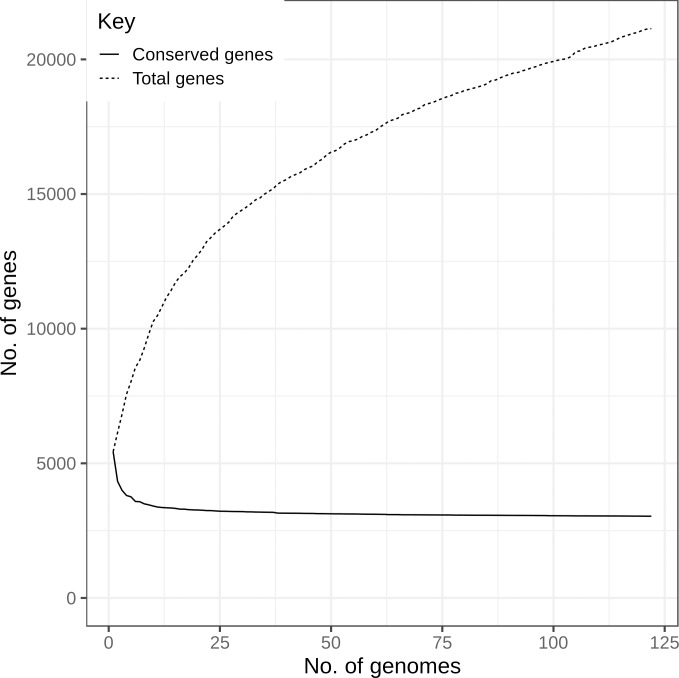
Comparison of the number of conserved and total genes in the serogroup O145 pangenome with increasing number of genomes. This analysis indicates the effect an increasing number of serogroup O145 genomes included in the analysis has on the number of conserved and total genes.

**Fig 5:**
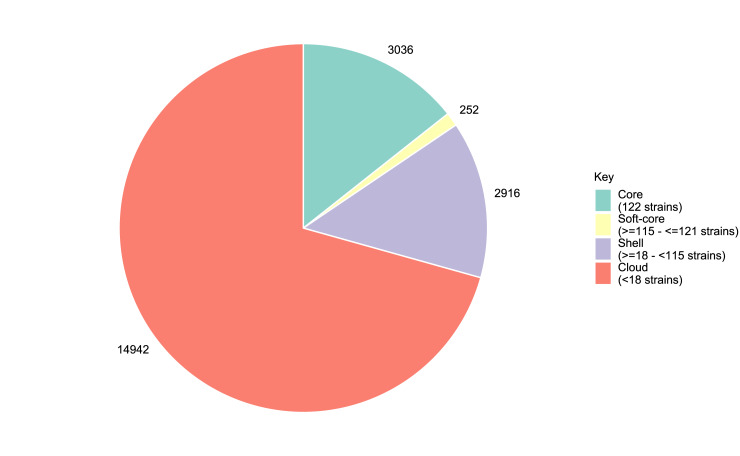
The pangenome composition of serogroup O145 strains (n = 122). Pan genome composition of serogroup O145 strains (n = 122) showing the core as genes present in all strains (100%), the soft-core as genes present in ≥115 and ≤121 strains (≥95%—≤99%), the shell as genes present in ≥18 - <115 strains (≥15%—<95%) and the cloud <18 strains (>0%—<15%).

The number of conserved and total genes present in serogroup O145 strains (n = 122) is shown in Fig 4. The core is defined as genes present in all strains (100%) strains, the soft-core as genes present in between ≥115 and ≤121 strains (≥95%—≤99%) strains, the shell as genes present in ≥18 - <115 strains (≥15%—<95%) strains and the cloud as genes present in <18 strains (>0%—<15%) (Fig 5). The pangenome analysis suggested a core gene set of 3,036 genes, a soft-core of 252 genes, a shell of 2,916 genes and a cloud of 14,942 genes (Fig 5).

Pangenome analysis of serogroup O145 strains (n = 122) supported the diverse genetic heterogeneity within this serogroup (Fig 5). For a given population, when additional genome sequences are included, an open pangenome will identify un-characterised genes, whereas a closed pangenome will have approached a constant number [85]. The serogroup O145 pangenome of >14,000 genes was open (Fig 4), demonstrating the genetic heterogeneity of this dataset, and is consistent with the identification of distinct phylogenetic lineages. The number of core genes reported for *E*. *coli* varies among studies and ranges from 1,472 to 5,173 [43, 44, 86–88]. Although pangenomes consisting of >13,000 genes have been reported for *E*. *coli* [86, 88], pangenome analysis of 325 *E*. *coli* O26 genome sequences and 239 O145:H28 strains indicated an open pangenome with an accessory genome of only 8,804 genes [88] and 9,342 [44], respectively. These core genome variations are likely to be due to factors such as the number of genomes analysed and the genetic similarity of the strains included for comparison. For example, analysis of two serogroup O145:H28 strains identified a large core gene set of 5,173 as the two strains are likely to be genetically very similar [43] and the core gene set of 239 O145:H28 strains was 3,804 [44]. In addition, different software and identity thresholds can be used to define pangenomes, for example, a study of 53 *E*. *coli* genomes identified a core genome of 1,472 when reporting gene families rather than individual genes [86]. Therefore, such parameters should be considered when comparing between studies. Pangenome analysis has indicated a significant proportion of the *E*. *coli* genome as comprised of diverse genes. In O145:H28 strains (n = 239), plasmid- and phage-associated genes comprised a large proportion of the pangenome [44]. This highlights the genetic heterogeneity of *E*. *coli* through HGT, incorporation of phage genetic material and through gene loss or duplication that can lead to genetically diverse populations, even within the same serogroup.

### Core SNP analysis

**Fig 6:**
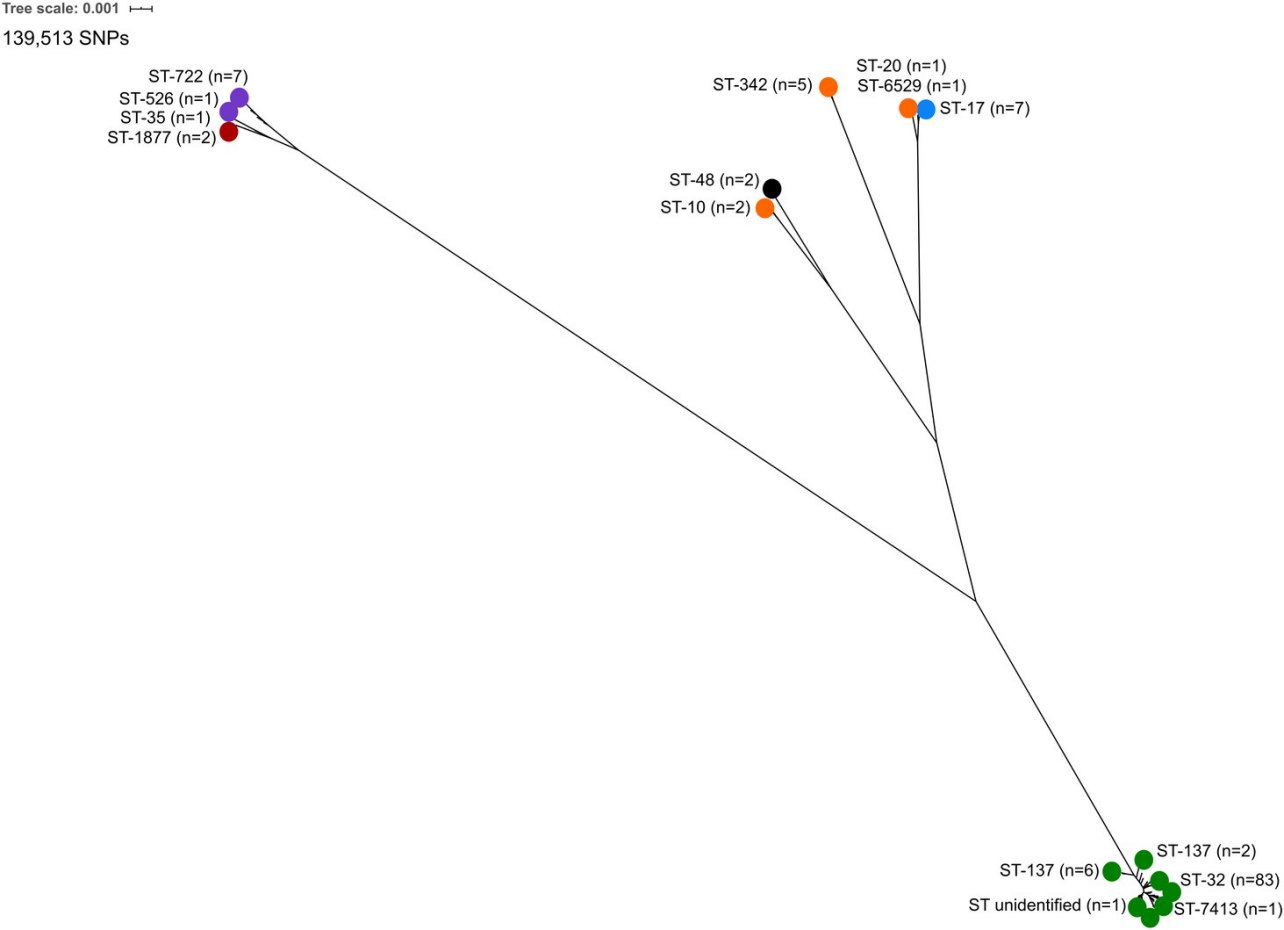
Maximum-likelihood tree of core single nucleotide polymorphism analysis of serogroup O145 strains (n = 122). RAxML Next-Generation maximum-likelihood tree of the core single nucleotide polymorphism (SNP) genome analysis from serogroup O145 genome sequences (n = 122). The tree was generated using 139,513 core SNPs. Metadata is included for *eae* subtype and sequence type, and additional information for each isolate can be found in S1 and S2 Tables.

Core SNP analysis of the 122 serogroup O145 strains (Fig 6) separated the strains into five phylogenetic clades which correlated with both *eae* subtype and ST. Clade 1 consisted of *eae* subtypes ι (n = 9) and α2 (n = 2). Clade 2 consisted of *eae* subtype β strains belonging to ST10 (n = 2) and the two *eae*-negative strains. *eae* subtype β strains belonging to ST342 (n = 5) formed Clade 3, whilst Clade 4 consisted of *eae* subtype ε strains (n = 7) and two *eae* subtype β strains belonging to ST20 and ST6529. The largest group, Clade 5, consisted of *eae* subtype γ strains (n = 93) and 139,513 SNPs were identified within the core genome of these strains (n = 122). Genome-wide core SNP analysis of 69 *E*. *coli* strains across 31 serogroups also identified significant genetic diversity with 86,350 SNPs identified across 1,371 core genes [42]. These results support the hypothesis of the evolution of distinct *E*. *coli* phylogenetic lineages with different *eae* subtypes, with subsequent mutations and/or HGT resulting in a large amount of genetic heterogeneity.

**Fig 7:**
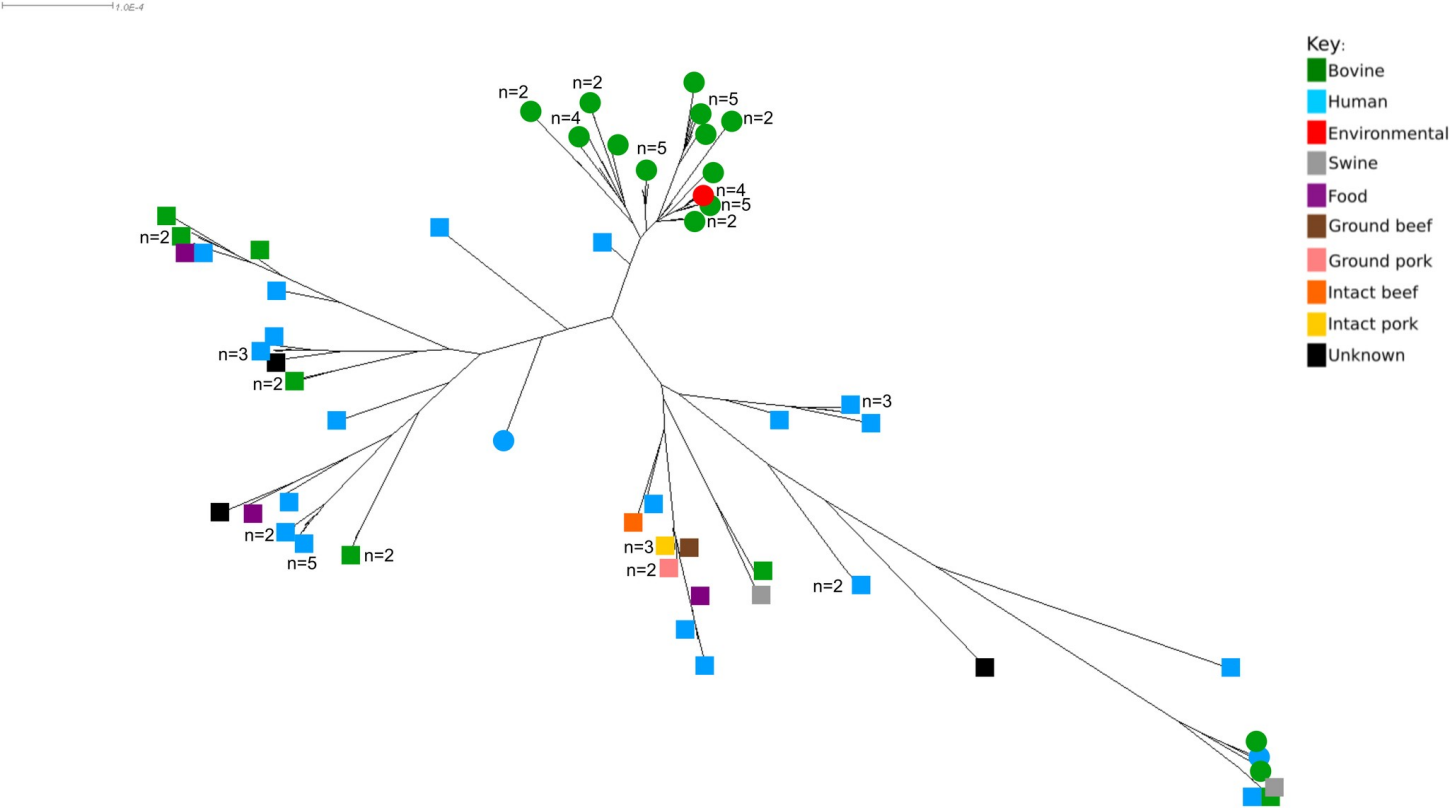
Maximum-likelihood tree of core single nucleotide polymorphism analysis from *eae* subtype γ serogroup O145 strains (n = 93). RAxML Next-Generation maximum-likelihood tree of the core single nucleotide polymorphism (SNP) genome analysis from serogroup O145 *eae* subtype γ genome sequences (n = 93). The tree was generated using 6,534 core SNPs. Circular and square nodes indicate genomes of New Zealand and non-New Zealand origin, respectively. Metadata is included for isolation source, and additional information for each isolate can be found in S1 and S2 Tables.

To resolve the phylogeny of the serogroup O145 strains, a core SNP analysis was performed on the *eae* subtype γ strains (n = 93) (Fig 7) identifying 6,534 SNPs, accounting for only 4.7% of the variation seen in the core genome of serogroup O145 strains (n = 122). This indicates these strains are genetically more similar compared to the other serogroup O145 strains analysed in this study.

### Utilisation of carbon substrates

**Fig 8:**
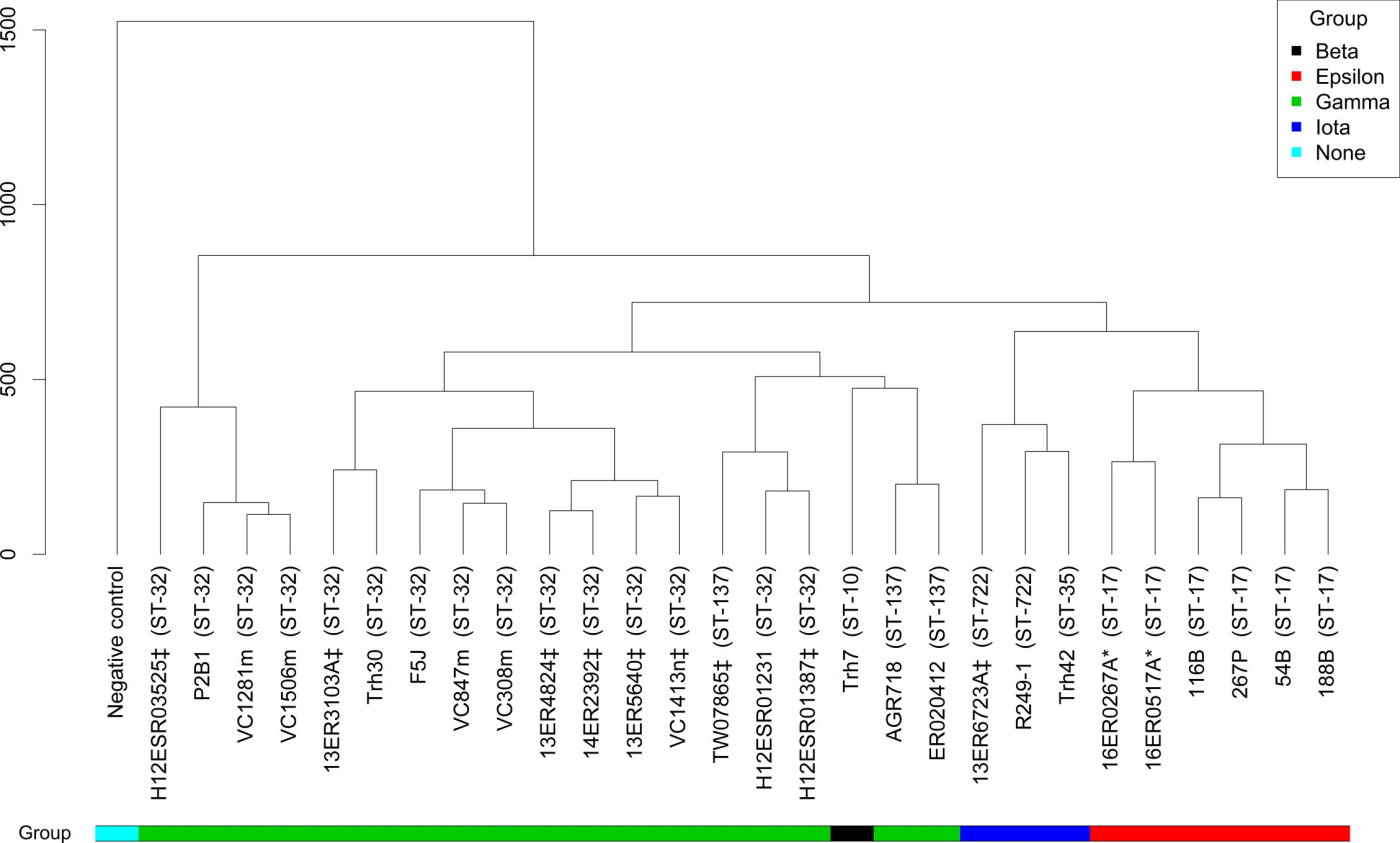
Cluster dendrogram showing the similarities of *E*. *coli* serogroup O145 strains based on their carbon utilisation profile using PM1 MicroPlate™. The end-point values per serogroup O145 strain (n = 28) for each carbon substrate on the phenotypic microarray plates (n = 95) was recorded and used to produce a cluster dendrogram using hierarchical clustering, with height indicating the distance between pairs. Metadata is included for sequence type, *eae* subtype and whether the strains were toxigenic or non-toxigenic. Sequence type is shown in brackets, *stx1* positive as * and *stx2* positive as ‡.

A dendrogram was generated according to the clustering of the utilisation of carbon substrates on the PM1 MicroPlates™, and significant metabolic variation was observed between serogroup O145 strains (Fig 8). There was no relationship between the utilisation of specific carbon substrates and whether a strain was *stx*-positive or *stx*-negative. Similarly, strains of human and bovine origin did not cluster together (Fig 8, S2 Fig). Instead, clustering of serogroup O145 strains by carbon utilisation was broadly associated with *eae* subtype and ST, which is consistent with the genomic analysis and highlights both the metabolic and genetic heterogeneity of this serogroup.

The utilisation of carbon substrates and subsequent cluster analysis of metabolic characteristics displayed by serogroup O145 strains (n = 20) on the PM2A MicroPlates™ (S3 Fig) was similar to that seen with the PM1 MicroPlates™. However, far fewer carbon substrates (23.2%; 22 out of 95) were utilised by ≥1 *E*. *coli* strain on the PM2A MicroPlates™. The clustering observed was similar when the replicate (analysed on separate days) and duplicate (analysed on the same day) data was included, however, there was contrasting utilisation of some carbon substrates between replicates and duplicates on the PM1 (11.6%; 11/95) and PM2A (4.2%; 4/95) MicroPlates™ (S2 and S3 Figs). Notably, the utilisation of some substrates, such as D-psicose and glucuronamide, was inconsistent between replicates and duplicates for multiple serogroup O145 strains, and due to the inconsistency in utilisation, these substrates are likely to be unsuitable for use in a differential media for serogroup O145.

Analysis of the utilisation of 190 carbon substrates (PM1 MicroPlates™, PM2A MicroPlates™) failed to identify any specific carbon substrates that would be likely to definitively differentiate serogroup O145 from other *E*. *coli*. However, several carbon substrates were identified which are utilised by a large proportion of serogroup O145 strains, which, when coupled with current molecular and culture-based methods, could aid in the identification of presumptive *E*. *coli* serogroup O145 isolates. For example, D-serine is utilised by *eae* subtype γ (ST32 and ST137; n = 18) and β (ST10; n = 1), and D-malic acid is utilised by *eae* subtypes γ (ST32 and ST137; n = 18) and ε (ST17; n = 6) (S2 Fig). These carbon substrates warrant subsequent testing with additional serogroup O145 strains and further non-O145 strains by including them as the main energy source in a minimal medium or a selective enrichment media.

However, there is variation in carbon substrate utilisation within serogroup O145 strains of the same ST and *eae* subtype. This suggests that the ability to metabolise certain substrates has either been lost or gained independently on multiple occasions by entirely separate lineages of serogroup O145. Furthermore, this also suggests that HGT events can lead to phenotypic traits that are not homogenous between members of the same phylogenetic cluster (either by MLST or SNP-based typing). Contrasting phenotypic traits leading to variations in metabolic activity may also arise via point mutations. Other traits, such as virulence factors and antimicrobial resistance, are also highly heterogeneous within the *E*. *coli* serogroup O145 strains studied, highlighting the limitations of making assumptions about isolates belonging to one serogroup from genetic data with limited phylogenetic resolution, such as the seven-gene MLST schemes. As a result, phenotypes cannot always be assumed from genotype, and therefore both phenotype and genotype testing are required to understand the epidemiological origin and potential virulence-associated consequences.

Previous studies have examined the carbon utilisation of the ‘Top 7’ *E*. *coli* serogroups [31, 89], demonstrating the variability in carbon utilisation and how this variability has hindered the development of a differential media for many non-O157 serogroups. However, this is the first study to examine the growth of genetically diverse serogroup O145 strains with a broad range of carbon substrates. In other studies undertaken to compare metabolic capabilities of several STEC serogroups, O145 isolates (n = 3) showed little variation in the number of carbon substrates utilised and β-hydroxy-butyric acid was identified as a candidate metabolite for differentiation of O145 from other clinically relevant STEC [31]. However, our study of genetically diverse O145 demonstrated that the utilisation of β-hydroxy-butyric acid was variable (S2 Fig) with some O145 (*eae* subtype β (n = 1), ε (n = 4), ι (n = 3) and γ (n = 1, ST137)) strains unable to utilise β-hydroxy-butyric acid as the only carbon source. The three O145 strains examined previously [31] displayed similarities in carbon utilisation with O157:H7 strains (*eae* subtype γ), were all O145:H28, and are likely to be *eae* subtype γ, which may account for the limited variation observed in comparison to the heterogeneity seen in our study [31]. In contrast, carbon utilisation of *E*. *coli* (n = 153), *Shigella* (n = 16), *Escherichia fergusonii* (n = 2), *Escherichia albertii* (n = 1) and cryptic *Escherichia* Clade strains (n = 6) in another study was shown to be highly variable [90]. The carbon substrate utilisation diversity observed in our study suggests the development of diagnostic media permitting the selective growth and/or differentiation of all O145 strains based on carbon source utilisation could be difficult. However, if the high prevalence of serogroup O145 *eae* subtype γ strains seen in this study (S1 and S2 Tables) is reflected in their overall zoonotic potential, the development of a medium solely for this *eae* subtype may be beneficial.

## Conclusion

In this study, we used comparative genomics and carbon substrate utilisation to understand the genomic epidemiology and metabolic profiles of *E*. *coli* serogroup O145, respectively. We found considerable genetic heterogeneity within serogroup O145 strains according to the relative abundance of virulence factors, core genome SNPs, and pangenome analysis. ST and *eae* subtype provided an indication of genetic heterogeneity suggesting these parameters are good indicators to separate distinct *E*. *coli* phylogenetic lineages. The genetic heterogeneity within these strains also provided evidence of a broad virulence continuum; *stx*2*a*- and *eae*-positive strains are implicated as the cause of severe human disease, both typical and atypical EPEC are associated with mild diarrhoeal disease or asymptomatic carriage, while other serogroup O145 isolated from wolves lacked many STEC-associated virulence factors and appeared to be host-associated and unlikely zoonoses. Carbon substrate utilisation by a subset of *E*. *coli* serogroup O145 strains demonstrated considerable metabolic variation, which showed a remarkable association with *eae* subtype and ST, consistent with the genomic data. Several carbon substrates, such as D-serine and D-malic acid, were identified which are utilised by many serogroup O145 strains including *eae* subtype γ, the predominant *eae* subtype identified in this study. These carbon substrates, coupled with molecular tests to detect O145-specific *wzx* and *wzy* gene sequences, could provide targets for further investigation in media to assist in the identification of presumptive *E*. *coli* serogroup O145 strains. Further testing with additional non-O145 isolates is required to test this hypothesis.

## Supporting information

S1 Table Bacterial strains whole genome sequenced in this study. (DOCX)

S2 Table Publicly available genome sequences analysed in this study. (DOCX)

S3 Table Accession numbers for *E*. *coli* serogroup O145 stains whole genome sequenced in this study. (DOCX)

S4 Table Presence and absence data for serogroup O145 genomes (n = 122) from 37 virulence genes identified by the center for genomic epidemiology VirulenceFinder webserver. (DOCX)

S5 Table Plasmid incompatibility factors identified from serogroup O145 whole genome sequence data (n = 122). (DOCX)

S6 Table *E*. *coli* tRNA integration site for the Locus for Enterocyte Effacement (LEE) pathogenicity island for serogroup O145 genomes (n = 120). (DOCX)

S1 Fig Box and whisker plots indicating the assembly statistics of *E*. *coli* serogroup O145 strains (n = 122). The box and whisker plots indicate the number of contigs, largest contig and N_50_ value for the serogroup O145 strains (n = 122). Each data point is shown on the plots and has been colour coded according to *eae* subtype, as indicated by the figure key.
(PNG)

S2 Fig Heat-map showing *E*. *coli* serogroup O145 strains carbon utilisation profiles using PM1 MicroPlate™ with replicates and duplicates. Heat-map of PM1 carbon substrate metabolism over a 24-hour incubation period at 37°C by serogroup O145 strains. The end-point utilisation values (Omnilog Units) were grouped into the following three categories: 0–50 representing no utilisation, 51–150 representing moderate utilisation and 151–400 representing extensive utilisation, as indicated by the colour key. Each strain (n = 28, n = 14 replicates, n = 2 duplicates) is indicated on the right and the 95 carbon substrates listed along at the foot of the figure. Metadata is included for *eae* subtype, sequence type, source and whether the strains were toxigenic. *eae* subtype on the left is represented by the colour key, NA is not applicable, sequence type is shown in brackets, isolate source indicated by the colour boxes next to the label name with black, red and blue boxes representing bovine, human and environmental sources, respectively and *stx1* positive as * and *stx2* positive as ‡.
(PNG)

S3 Fig Heat-map showing *E*. *coli* serogroup O145 strains carbon utilisation profiles using PM2A MicroPlate™ with replicates. Heat-map of PM2A carbon substrate metabolism over a 24-hour incubation period at 37°C by serogroup O145 strains. The end-point utilisation values (Omnilog Units) were grouped into the following three categories: 0**–**50 representing no utilisation, 51**–**150 representing moderate utilisation and 151**–**400 representing extensive utilisation, as indicated by the colour key. Each strain (n **=** 20 and n **=** 4 replicates) is indicated on the right and the 95 carbon substrates listed along at the foot of the figure. Metadata is included for *eae* subtype, sequence type, source and whether the strains were toxigenic. *eae* subtype on the left is represented by the colour key, NA is not applicable, sequence type is shown in brackets, isolate source indicated by the colour boxes next to the label name with black, red and blue boxes representing bovine, human and environmental sources, respectively *stx1* positive as * and *stx2* positive as ‡.
(PNG)
