# Supplementary material for: Genomic epidemiology and carbon metabolism of Escherichia coli serogroup O145 reflect contrasting phylogenies
Source: PLoS One. 2020 Jun 25;15(6):e0235066. doi: 10.1371/journal.pone.0235066 (PMC7316241; doi:10.1371/journal.pone.0235066)
Supplement: S1 Table — (DOCX) [file pone.0235066.s001.docx]

## Table S1: Bacterial strains whole genome sequenced in this study

| **Strain** | **Serotype** | **Source** | **Origin** | **Virulence profile** | ***eae* subtype** | **Sequence type** | **Reference/ source** |
| --- | --- | --- | --- | --- | --- | --- | --- |
| 116B | O145:H2 | Bovine | Taranaki, New Zealand | *eae, ehxA* | ε | ST-17 | Irshad (2013) |
| 13ER3103A | O145:H28 | Human | Auckland, New Zealand | *stx2, eae, ehxA* | γ | ST-32 | The Institute of Environmental Science and Research Ltd (n.d.) |
| 13ER4824 | O145:H28 | Bovine | New Zealand | *stx2, eae, ehxA* | γ | ST-32 | The Institute of Environmental Science and Research Ltd (n.d.) |
| 13ER5056 | O145:H28 | Bovine | New Zealand | *stx2, eae, ehxA* | γ | ST-32 | The Institute of Environmental Science and Research Ltd (n.d.) |
| 13ER5154 | O145:H28 | Bovine | New Zealand | *stx2, eae, ehxA* | γ | ST-32 | The Institute of Environmental Science and Research Ltd (n.d.) |
| 13ER5640 | O145:H28 | Bovine | New Zealand | *stx2, eae, ehxA* | γ | ST-32 | The Institute of Environmental Science and Research Ltd (n.d.) |
| 13ER6227 | O145:H28 | Bovine | New Zealand | *eae, ehxA* | γ | ST-32 | The Institute of Environmental Science and Research Ltd (n.d.) |
| 13ER6723A | O145:H34 | Human | Auckland, New Zealand | *stx2, eae* | ι | ST-722 | The Institute of Environmental Science and Research Ltd (n.d.) |
| 14ER2392 | O145:H28 | Bovine | New Zealand | *stx2, eae, ehxA* | γ | ST-32 | The Institute of Environmental Science and Research Ltd (n.d.) |
| 15ER2679 | O145:H28 | Bovine | New Zealand | *eae, ehxA* | γ | ST-32 | The Institute of Environmental Science and Research Ltd (n.d.) |
| 16ER0267A | O145:H2 | Human | Auckland, New Zealand | *stx1, eae, ehxA* | ε | ST-17 | The Institute of Environmental Science and Research Ltd (n.d.) |
| 16ER0517A | O145:H2 | Human | Auckland, New Zealand | *stx1, eae, ehxA* | ε | ST-17 | The Institute of Environmental Science and Research Ltd (n.d.) |
| 188B | O145:H2 | Bovine | Taranaki, New Zealand | *eae, ehxA* | ε | ST-17 | Irshad (2013) |
| 267P | O145:H2 | Bovine | Taranaki, New Zealand | *eae, ehxA* | ε | ST-17 | Irshad (2013) |
| 54B | O145:H2 | Bovine | Taranaki, New Zealand | *eae, ehxA* | ε | ST-17 | Irshad (2013) |
| AGR718 | O145:H46 | Bovine | Manawatu, New Zealand | *eae, ehxA* | γ | ST-137 | Cookson et al. (2010) |
| ERL020412 | O145:H28 | Human | New Zealand | *eae, ehxA* | γ | ST-137 | The Institute of Environmental Science and Research Ltd (n.d.) |
| ERL121829 | O145:H28 | Bovine | New Zealand | *eae, ehxA* | γ | ST-32 | The Institute of Environmental Science and Research Ltd (n.d.) |
| ERL122034 | O145:H2 | Bovine | New Zealand | *eae, ehxA* | ε | ST-17 | The Institute of Environmental Science and Research Ltd (n.d.) |
| F1 | O145:H28 | Environmental | Waikato, New Zealand | *eae, ehxA* | γ | ST-32 | Ross, Rapp, and Brightwell (2017) |
| F5F | O145:H28 | Environmental | Waikato, New Zealand | *eae, ehxA* | γ | ST-32 | Ross et al. (2017) |
| F5J | O145:H28 | Environmental | Waikato, New Zealand | *eae, ehxA* | γ | ST-32 | Ross et al. (2017) |
| FDE21 | O145:H28 | Environmental | Waikato, New Zealand | *eae, ehxA* | γ | ST-32 | Ross et al. (2017) |
| H12ESR01231 | O145:H28 | Bovine | New Zealand | *eae, ehxA* | γ | ST-32 | The Institute of Environmental Science and Research Ltd (n.d.) |
| H12ESR01387 | O145:H28 | Bovine | New Zealand | *stx2, eae, ehxA* | γ | ST-32 | The Institute of Environmental Science and Research Ltd (n.d.) |
| H12ESR01650 | O145:H28 | Bovine | New Zealand | *eae, ehxA* | γ | ST-32 | The Institute of Environmental Science and Research Ltd (n.d.) |
| H12ESR03525 | O145:H28 | Bovine | New Zealand | *stx2, eae, ehxA* | γ | ST-32 | The Institute of Environmental Science and Research Ltd (n.d.) |
| P2A1 | O145:H28 | Environmental | Waikato, New Zealand | *eae, ehxA* | γ | ST-32 | Ross et al. (2017) |
| P2B1 | O145:H28 | Environmental | Waikato, New Zealand | *eae, ehxA* | γ | ST-32 | Ross et al. (2017) |
| R249-1 | O145:H34 | Human | Australia | *eae* | ι | ST-722 | Nguyen, Taylor, Tauschek, and Robins-Browne (2006) |
| Trh30 | O145:H28 | Human | Norway | *eae, ehxA* | γ | ST-32 | Afset et al. (2008) |
| Trh42 | O145:H34 | Human | Norway | *eae* | ι | ST-35 | Afset et al. (2008) |
| Trh46 | O145:H34 | Human | Norway | *eae* | ι | ST-526 | Afset et al. (2008) |
| Trh7 | O145:H40 | Human | Norway | *eae* | β | ST-10 | Afset et al. (2008) |
| TW07865 | O145:H28 | Human | Germany | *stx2, eae, ehxA* | γ | ST-137 | The Institute of Environmental Science and Research Ltd (n.d.) |
| VC1048m | O145:H28 | Bovine | Taranaki, New Zealand | *eae, ehxA* | γ | ST-137 | This study |
| VC1056m | O145:H28 | Bovine | Taranaki, New Zealand | *eae, ehxA* | γ | ST-32 | This study |
| VC123n | O145:H28 | Bovine | Waikato, New Zealand | *eae, ehxA* | γ | ST-32 | This study |
| VC1281m | O145:H28 | Bovine | Canterbury, New Zealand | *eae, ehxA* | γ | ST-32 | Browne et al. (2018) |
| VC1413m | O145:H28 | Bovine | Southland, New Zealand | *stx2, eae, ehxA* | γ | ST-32 | This study |
| VC1506m | O145:H28 | Bovine | Southland, New Zealand | *eae, ehxA* | γ | ST-32 | Browne et al. (2018) |
| VC194m | O145:H28 | Bovine | Northland, New Zealand | *eae, ehxA* | γ | ST-32 | This study |
| VC237m | O145:H28 | Bovine | Northland, New Zealand | *eae, ehxA* | γ | ST-32 | This study |
| VC237o | O145:H28 | Bovine | Northland, New Zealand | *eae, ehxA* | γ | ST-32 | This study |
| VC308m | O145:H28 | Bovine | Northland, New Zealand | *eae, ehxA* | γ | ST-32 | This study |
| VC476m | O145:H28 | Bovine | Waikato, New Zealand | *eae, ehxA* | γ | Unknown | This study |
| VC506m | O145:H28 | Bovine | Waikato, New Zealand | *eae, ehxA* | γ | ST-32 | This study |
| VC525m | O145:H28 | Bovine | Waikato, New Zealand | *eae, ehxA* | γ | ST-32 | Browne et al. (2018) |
| VC554m | O145:H28 | Bovine | Waikato, New Zealand | *eae, ehxA* | γ | ST-32 | This study |
| VC847m | O145:H28 | Bovine | Manawatu-Wellington, New Zealand | *eae, ehxA* | γ | ST-32 | Browne et al. (2018) |
| VC849m | O145:H28 | Bovine | Manawatu-Wellington, New Zealand | *eae, ehxA* | γ | ST-32 | This study |
| VC874o | O145:H28 | Bovine | Taranaki, New Zealand | *eae, ehxA* | γ | ST-32 | Browne et al. (2018) |
| VC880m | O145:H28 | Bovine | Taranaki, New Zealand | *eae, ehxA* | γ | ST-32 | This study |
