# Supplementary material for: Genomic epidemiology and carbon metabolism of Escherichia coli serogroup O145 reflect contrasting phylogenies
Source: PLoS One. 2020 Jun 25;15(6):e0235066. doi: 10.1371/journal.pone.0235066 (PMC7316241; doi:10.1371/journal.pone.0235066)
Supplement: S2 Table — (DOCX) [file pone.0235066.s002.docx]

**Table S2: Publicly available genome sequences analysed in this study**

| **Isolate** | **Serotype** | **Source** | **Origin** | **STEC** | ***eae* subtype** | **Sequence type** | **SRA accession number** | **BioSample ID** | **Reference/ source** |
| --- | --- | --- | --- | --- | --- | --- | --- | --- | --- |
| 073858 | O145:H25 | Human | USA | + | β | ST-342 | SRR6373675 | SAMN02352916 | Lindsey et al. (2014) |
| 130322 | O145:H28 | Human | UK | + | γ | ST-32 | SRR3579383 | [SAMN05171053](https://www.ncbi.nlm.nih.gov/biosample/SAMN05171053) | Public Health England (2016) |
| 132030 | O145:H28 | Human | UK | + | γ | ST-32 | SRR3578591 | [SAMN05170684](https://www.ncbi.nlm.nih.gov/biosample/SAMN05170684) | Public Health England (2016) |
| 143974 | O145:H34 | Human | UK | + | ι | ST-722 | SRR4176975 | SAMN05733905 | Health Protection Agency. (2016) |
| 170303 | O145:H34 | Human | UK | - | ι | ST-722 | SRR3578794 | [SAMN05170850](https://www.ncbi.nlm.nih.gov/biosample/SAMN05170850) | Public Health England (2016) |
| 173582 | O145:H28 | Human | UK | + | γ | ST-32 | SRR3581442 | [SAMN05171900](https://www.ncbi.nlm.nih.gov/biosample/SAMN05171900) | Public Health England (2016) |
| 173758 | O145:H28 | Human | UK | + | γ | ST-32 | SRR3581328 | [SAMN05171781](https://www.ncbi.nlm.nih.gov/biosample/SAMN05171781) | Public Health England (2016) |
| 182131 | O145:H40 | Human | UK | - | β | ST-10 | SRR3581355 | [SAMN05171820](https://www.ncbi.nlm.nih.gov/biosample/SAMN05171820) | Public Health England (2016) |
| 199816 | O145:H34 | Human | UK | - | α2 | ST-722 | SRR3578986 | [SAMN05171019](https://www.ncbi.nlm.nih.gov/biosample/SAMN05171019) | Public Health England (2016) |
| 2009C-3292 | O145:H25 | Unknown | USA | + | β | ST-342 | SRR3188742 | SAMN04516766 | Trees et al. (2014) |
| 2010C-3507 | O145:H28 | Human | USA | + | γ | ST-32 | SRR3371785 | [SAMN02352965](https://www.ncbi.nlm.nih.gov/biosample/SAMN02352965) | Trees et al. (2014) |
| 2010C-3508 | O145:H28 | Human | USA | + | γ | ST-32 | SRR3371786 | [SAMN02352966](https://www.ncbi.nlm.nih.gov/biosample/SAMN02352966) | Trees et al. (2014) |
| 2010C-3509 | O145:H28 | Human | USA | + | γ | ST-32 | SRR3371787 | [SAMN02352967](https://www.ncbi.nlm.nih.gov/biosample/SAMN02352967) | Trees et al. (2014) |
| 2010C-3510 | O145:H28 | Human | USA | + | γ | ST-32 | SRR3371788 | [SAMN02352968](https://www.ncbi.nlm.nih.gov/biosample/SAMN02352968) | Trees et al. (2014) |
| 2010C-3526 | O145:H28 | Human | USA | + | γ | ST-32 | SRR3371794 | [SAMN02352974](https://www.ncbi.nlm.nih.gov/biosample/SAMN02352974) | Trees et al. (2014) |
| 2012C-4474 | O145:H28 | Human | USA | + | γ | ST-32 | SRR975374 | [SAMN02352667](https://www.ncbi.nlm.nih.gov/biosample/SAMN02352667) | FDA Center for Food Safety and Applied Nutrition (2013) |
| 2012C-4477 | O145:H28 | Human | USA | + | γ | ST-32 | SRR975375 | [SAMN02352668](https://www.ncbi.nlm.nih.gov/biosample/SAMN02352668) | FDA Center for Food Safety and Applied Nutrition (2013) |
| 2012C-4478 | O145:H28 | Human | USA | + | γ | ST-32 | SRR975376 | [SAMN02352669](https://www.ncbi.nlm.nih.gov/biosample/SAMN02352669) | FDA Center for Food Safety and Applied Nutrition (2013) |
| 2012C-4479 | O145:H28 | Human | USA | + | γ | ST-32 | SRR975377 | [SAMN02352670](https://www.ncbi.nlm.nih.gov/biosample/SAMN02352670) | FDA Center for Food Safety and Applied Nutrition (2013) |
| 201499 | O145:H34 | Human | UK | - | α2 | ST1877 | SRR3578586 | [SAMN05170678](https://www.ncbi.nlm.nih.gov/biosample/SAMN05170678) | Public Health England (2016) |
| 203972 | O145:H34 | Human | UK | - | ι | ST-722 | SRR4192081 | [SAMN05750680](https://www.ncbi.nlm.nih.gov/biosample/SAMN05750680) | Public Health England (2016) |
| 238454 | O145:H28 | Human | UK | - | ϒ | ST-32 | SRR3574222 | SAMN05163699 | Health Protection Agency. (2016) |
| 241761 | O145:H28 | Human | UK | + | γ | ST-32 | SRR3574267 | [SAMN05163744](https://www.ncbi.nlm.nih.gov/biosample/SAMN05163744) | Public Health England (2016) |
| 241810 | O145:H28 | Human | UK | - | γ | ST-32 | SRR3574240 | [SAMN05163717](https://www.ncbi.nlm.nih.gov/biosample/SAMN05163717) | Public Health England (2016) |
| 82EZXG | O145:H28 | Human | Scotland | + | ϒ | ST-32 | SRR6321364 | SAMN08095912 | NHS Lothian. (2017) |
| AA053 | O145:H28 | Human | Denmark | - | γ | ST-137 | ERR1010242 | [SAMEA3529328](https://www.ncbi.nlm.nih.gov/biosample/SAMEA3529328) | Joensen, Tetzschner, Iguchi, Aarestrup, and Scheutz (2015) |
| BCW4180 | O145:H28 | Unknown | Unknown | - | ϒ | ST-137 | SRR1118462 | SAMN02368911 | University of California at Davis. (2016) |
| BYSO3C | O145:H34 | Human | Scotland | + | ι | ST-722 | SRR6321368 | SAMN08095908 | NHS Lothian. (2017) |
| ED657 | O145:H28 | Human | Italy | + | ϒ | ST-32 | ERR2365411 | SAMEA3677960 | Istituto Superiore Di Sanita. (2016) |
| FSIS1400369 | O145:H28 | Bovine | USA | + | γ | ST-32 | SRR1693413 | [SAMN03216751](https://www.ncbi.nlm.nih.gov/biosample/SAMN03216751) | U.S Department of Agriculture (2014) |
| FSIS1500788 | O145:HNT | Intact beef | USA | - | γ | ST-32 | SRR2126002 | [SAMN03922108](https://www.ncbi.nlm.nih.gov/biosample/SAMN03922108) | U.S Department of Agriculture (2015) |
| FSIS1500875 | O145:H28 | Food | USA | + | ϒ | ST-32 | SRR2125830 | SAMN03921927 | FDA Center for Food Safety and Applied Nutrition (2014) |
| FSIS1501198 | O145:H28 | Bovine | USA | + | γ | ST-32 | SRR3405428 | [SAMN04870300](https://www.ncbi.nlm.nih.gov/biosample/SAMN04870300) | U.S Department of Agriculture (2016) |
| FSIS1501717 | O145:H28 | Ground beef | USA | + | γ | ST-32 | SRR3405608 | [SAMN04870303](https://www.ncbi.nlm.nih.gov/biosample/SAMN04870303) | U.S Department of Agriculture (2016) |
| FSIS1502535 | O145:H28 | Intact pork | USA | + | γ | ST-32 | SRR3185246 | [SAMN04510513](https://www.ncbi.nlm.nih.gov/biosample/SAMN04510513) | U.S Department of Agriculture (2016) |
| FSIS1502550 | O145:H28 | Intact pork | USA | + | γ | ST-32 | SRR3175216 | [SAMN04497383](https://www.ncbi.nlm.nih.gov/biosample/SAMN04497383) | U.S Department of Agriculture (2016) |
| FSIS1502554 | O145:H28 | Intact pork | USA | + | γ | ST-32 | SRR3175217 | [SAMN04497385](https://www.ncbi.nlm.nih.gov/biosample/SAMN04497385) | U.S Department of Agriculture (2016) |
| FSIS1502976 | O145:H28 | Ground pork | USA | + | γ | ST-32 | SRR3175218 | [SAMN04497386](https://www.ncbi.nlm.nih.gov/biosample/SAMN04497386) | U.S Department of Agriculture (2016) |
| FSIS1502978 | O145:H28 | Ground pork | USA | + | γ | ST-32 | SRR3185253 | [SAMN04510516](https://www.ncbi.nlm.nih.gov/biosample/SAMN04510516) | U.S Department of Agriculture (2016) |
| FSIS1503305 | O145:H28 | Bovine | USA | + | γ | ST-32 | SRR3441262 | [SAMN04901729](https://www.ncbi.nlm.nih.gov/biosample/SAMN04901729) | U.S Department of Agriculture (2016) |
| FSIS1503307 | O145:H28 | Bovine | USA | + | γ | ST-32 | SRR3441301 | [SAMN04901731](https://www.ncbi.nlm.nih.gov/biosample/SAMN04901731) | U.S Department of Agriculture (2016) |
| FSIS1504619 | O145 | Bovine | USA | + | γ | ST-32 | SRR2826835 | [SAMN04208149](https://www.ncbi.nlm.nih.gov/biosample/SAMN04208149) | U.S Department of Agriculture (2015) |
| FSIS1505314 | O145 | Bovine | USA | + | γ | ST-32 | SRR3106214 | [SAMN04421068](https://www.ncbi.nlm.nih.gov/biosample/SAMN04421068) | U.S Department of Agriculture (2016) |
| FSIS1605419 | O145 | Bovine | USA | + | γ | ST-32 | SRR3106215 | [SAMN04421070](https://www.ncbi.nlm.nih.gov/biosample/SAMN04421070) | U.S Department of Agriculture (2016) |
| FSIS1605420 | O145 | Bovine | USA | + | γ | ST-32 | SRR3106213 | [SAMN04421071](https://www.ncbi.nlm.nih.gov/biosample/SAMN04421071) | U.S Department of Agriculture (2016) |
| FSIS1605733 | O145:H28 | Food | USA | + | ϒ | ST-32 | SRR3185249 | SAMN04510518 | FDA Center for Food Safety and Applied Nutrition (2014) |
| FSIS1700607 | O145:H28 | Food | USA | + | ϒ | ST-7413 | SRR5589518 | SAMN07159181 | FDA Center for Food Safety and Applied Nutrition (2014) |
| MOD1EC1641 | O145:H28 | Human | USA | + | ϒ | ST-32 | SRR4340479 | SAMN05607397 | Gangiredla et al. (2017) |
| MOD1EC1661 | O145:H28 | Human | USA | + | ϒ | ST-32 | SRR4340493 | SAMN05607381 | Gangiredla et al. (2017) |
| MOD1EC1672 | O145:H25 | Human | Uruguay | + | β | ST-342 | SRR5330931 | SAMN05607372 | Gangiredla et al. (2017) |
| MOD1EC1935 | O145:H28 | Bovine | USA | + | ϒ | ST-32 | SRR4340514 | SAMN05605301 | Gangiredla et al. (2017) |
| MOD1EC1941 | O145:H25 | Bovine | USA | - | β | ST-342 | SRR4340522 | SAMN05605297 | Gangiredla et al. (2017) |
| MOD1EC1954 | O145:H28 | Bovine | Germany | + | ϒ | ST-32 | SRR4340532 | SAMN05605286 | Gangiredla et al. (2017) |
| MOD1EC1969 | O145:H28 | Human | USA | + | ϒ | ST-32 | SRR5330937 | SAMN05605310 | Gangiredla et al. (2017) |
| MOD1EC1971 | O145:H28 | Human | USA | + | ϒ | ST-32 | SRR5330939 | SAMN05605309 | Gangiredla et al. (2017) |
| MOD1EC1972 | O145:H28 | Human | USA | + | ϒ | ST-32 | SRR5330940 | SAMN05605358 | Gangiredla et al. (2017) |
| MOD1EC2002 | O145 | Human | USA | + | γ | ST-32 | SRR3124089 | [SAMN04256127](https://www.ncbi.nlm.nih.gov/biosample/SAMN04256127) | FDA Center for Food Safety and Applied Nutrition (2016) |
| MOD1EC5078 | O145:H11 | Wolf | USA | - | Negative | ST-48 | SRR3407781 | SAMN04279415 | Gangiredla et al. (2017) |
| MOD1EC5081 | O145:H11 | Wolf | USA | - | Negative | ST-48 | SRR3407784 | SAMN04279418 | Gangiredla et al. (2017) |
| MOD1EC5165 | O145:H2 | Unknown | Unknown | - | β | ST-20 | SRR3466382 | SAMN04279504 | Gangiredla et al. (2017) |
| MOD1EC5842 | O145 | Swine | USA | - | γ | ST-32 | SRR3987499 | [SAMN05439376](https://www.ncbi.nlm.nih.gov/biosample/SAMN05439376) | FDA Center for Food Safety and Applied Nutrition (2016) |
| MOD1EC5961 | O145:H2 | Swine | USA | - | β | ST-6529 | SRR3987718 | SAMN05439401 | Gangiredla et al. (2017) |
| MOD1EC6028 | O145 | Swine | USA | + | γ | ST-137 | SRR3988027 | [SAMN05439480](https://www.ncbi.nlm.nih.gov/biosample/SAMN05439480) | FDA Center for Food Safety and Applied Nutrition (2016) |
| MOD1EC6710 | O145:H25 | Bovine | USA | - | β | ST-342 | SRR3987664 | SAMN04992540 | Gangiredla et al. (2017) |
| OLC0719 | O145:H28 | Unknown | Canada | + | ϒ | ST-32 | SRR6154939 | SAMN05504943 | Knowles et al. (2016) |
| OLC1258 | O145:H34 | Unknown | Canada | + | ι | ST-722 | SRR6061315 | SAMN04420176 | Canadian Food Inspection Agency. (2017) |
| PNUSAE000756 | O145 | Human | USA | + | γ | ST-32 | SRR3541143 | [SAMN03782146](https://www.ncbi.nlm.nih.gov/biosample/SAMN03782146) | Centers for Disease Control and Prevention Enteric Diseases Laboratory Branch (2016) |
| PNUSAE001244 | O145 | Human | USA | + | γ | ST-32 | SRR2177987 | [SAMN04002944](https://www.ncbi.nlm.nih.gov/biosample/SAMN04002944) | Centers for Disease Control and Prevention Enteric Diseases Laboratory Branch (2016) |
| PNUSAE003232 | O145:H28 | Unknown | USA | + | ϒ | ST-32 | SRR3676339 | SAMN05225329 | Centers for Disease Control and Prevention Enteric Diseases Laboratory Branch (2014) |

Canadian Food Inspection Agency. (2017). *Application of genomics in the determination of verotoxigenic Escherichia coli toxin subtypes* [Raw sequence reads]. Retrieved from: <https://www.ncbi.nlm.nih.gov/bioproject/309770>

Centers for Disease Control and Prevention Enteric Diseases Laboratory Branch. (2014). *PulseNet Escherichia coli and Shigella genome sequencing* [Raw sequence reads]. Retrieved from: <https://www.ncbi.nlm.nih.gov/bioproject/218110>

Centers for Disease Control and Prevention Enteric Diseases Laboratory Branch. (2016). *PulseNet Escherichia coli and Shigella genome sequencing* [Whole genome sequences]. Retrieved from: <https://www.ncbi.nlm.nih.gov/sra/?term=SRR3371785>

FDA Center for Food Safety and Applied Nutrition. (2013). *Foodborne pathogen survey* [Whole genome sequences]. Retrieved from: <https://www.ncbi.nlm.nih.gov/sra/?term=SRR975374>

FDA Center for Food Safety and Applied Nutrition. (2014). *GenomeTrakr Project* [Whole genome sequences]. Retrieved from: <https://www.ncbi.nlm.nih.gov/sra/?term=SRR1272534>

FDA Center for Food Safety and Applied Nutrition. (2016). *Genome Trakr project: U.S Food and Drug Adminstration* [Whole genome sequences]. Retrieved from: <https://www.ncbi.nlm.nih.gov/sra/?term=SRR3124089>

Gangiredla, J., Mammel, M. K., Barnaba, T. J., Tartera, C., Gebru, S. T., Patel, I. R., . . . Lacher, D. W. (2017). Species-wide collection of *Escherichia coli* isolates for examination of genomic diversity. *Genome Announcements, 5*(50), e01321-01317. doi:10.1128/genomeA.01321-17

Health Protection Agency. (2016). *Routine surveillance of E. coli and Shigella by Public Health England* [Raw sequence reads]. Retrieved from: <https://www.ncbi.nlm.nih.gov/bioproject/315192>

Istituto Superiore Di Sanita. (2016). *Comparison of high resolution viruelnce allelic profiling (HReVAP) typing with multilocus sequence typing and whole genome SNPs analysis for typing VTEC strains* [Raw sequence reads]. Retrieved from: <https://www.ncbi.nlm.nih.gov/bioproject/340095>

Joensen, K. G., Tetzschner, A. M. M., Iguchi, A., Aarestrup, F. M., & Scheutz, F. (2015). Rapid and easy *in silico* serotyping of *Escherichia coli* isolates by use of whole genome sequencing data. *Journal of Clinical Microbiology, 53*(8), 2410-2426. doi:10.1128/jcm.00008-15

Knowles, M., Stinson, S., Lambert, D., Carrillo, C., Koziol, A., Gauthier, M., & Blais, B. (2016). Genomic tools for customized recovery and detection of foodborne Shiga toxigenic *Escherichia coli*. *Journal of Food Protection, 79*(12), 2066-2077. doi:10.4315/0362-028x.Jfp-16-220

Lindsey, R. L., Trees, E., Sammons, S., Loparev, V., Frace, M., Strockbine, N., . . . Gerner-Smidt, P. (2014). Draft whole-genome sequences of nine non-O157 Shiga toxin-producing *Escherichia coli* strains. *Genome Announcements, 2*(4), e00501-00514. doi:10.1128/genomeA.00501-14

NHS Lothian. (2017). *Whole genome sequencing of Shiga toxin-producing E. coli* [Raw sequence reads]. Retrieved from: <https://www.ncbi.nlm.nih.gov/bioproject?LinkName=biosample_bioproject&from_uid=8095912>

Public Health England. (2016). *Routine surveillance of E. coli and Shigella* [Whole genome sequences]. Retrieved from: <https://www.ncbi.nlm.nih.gov/sra/?term=SRR3581355>

Trees, E., Strockbine, N., Changayil, S., Ranganathan, S., Zhao, K., Weil, R., . . . Gerner-Smidt, P. (2014). Genome sequences of 228 Shiga toxin-producing *Escherichia coli* isolates and 12 isolates representing other diarrheagenic *E. coli* pathotypes. *Genome Announcements, 2*(4), e00718-00714.

U.S Department of Agriculture, F. S. a. I. S. (2014). *GenomeTrakr project: USDA-FSIS* [Whole genome sequences]. Retrieved from: <https://www.ncbi.nlm.nih.gov/sra/?term=SRR1693413>

U.S Department of Agriculture, F. S. a. I. S. (2015). *GenomeTrakr Project: USDA-FSIS* [Whole genome sequences]. Retrieved from: <https://www.ncbi.nlm.nih.gov/sra/?term=SRR2126002>

U.S Department of Agriculture, F. S. a. I. S. (2016). *GenomeTrakr project: USDA-FSIS* [Whole genome sequences]. Retrieved from: <https://www.ncbi.nlm.nih.gov/sra/?term=SRR3185246>

University of California at Davis. (2016). *Major food bacterial pathogens in the United States and around the world, including Salmonella enterica, E. coli, Shigella, Listeria monocytogenes, Campylobacter Jejuni, and Vibrio parahaemolyticus genome sequencing* [Raw sequence reads]. Retrieved from: <https://www.ncbi.nlm.nih.gov/bioproject?LinkName=biosample_bioproject&from_uid=2368911>
