# Supplementary material for: Genomic epidemiology and carbon metabolism of Escherichia coli serogroup O145 reflect contrasting phylogenies
Source: PLoS One. 2020 Jun 25;15(6):e0235066. doi: 10.1371/journal.pone.0235066 (PMC7316241; doi:10.1371/journal.pone.0235066)
Supplement: S3 Table — (DOCX) [file pone.0235066.s003.docx]

**Table S3: Accession numbers for *E. coli* serogroup O145 stains whole genome sequenced in this study**

| **Strain** | **BioProject** | **BioSample** | **Genome accession** | **SRA accession** |
| --- | --- | --- | --- | --- |
| VC880m | PRJNA435641 | SAMN08660766 | PXFW00000000 | SRR6951355 |
| VC874o | PRJNA435641 | SAMN08660749 | PXFX00000000 | SRR6951360 |
| VC849m | PRJNA435641 | SAMN08660765 | PXFY00000000 | SRR6951336 |
| VC847m | PRJNA435641 | SAMN08660746 | PXFZ00000000 | SRR6951357 |
| VC554m | PRJNA435641 | SAMN08660759 | PXGA00000000 | SRR6951332 |
| VC525m | PRJNA435641 | SAMN08660748 | PXGB00000000 | SRR6951359 |
| VC506m | PRJNA435641 | SAMN08660763 | PXGC00000000 | SRR6951328 |
| VC476m | PRJNA435641 | SAMN08660764 | PXGD00000000 | SRR6951337 |
| VC308m | PRJNA435641 | SAMN08660762 | PXGE00000000 | SRR6951329 |
| VC237o | PRJNA435641 | SAMN08660761 | PXGF00000000 | SRR6951326 |
| VC237m | PRJNA435641 | SAMN08660760 | PXGG00000000 | SRR6951327 |
| VC194m | PRJNA435641 | SAMN08660755 | PXGH00000000 | SRR6951350 |
| VC1506m | PRJNA435641 | SAMN08660750 | PXGI00000000 | SRR6951361 |
| VC1413m | PRJNA435641 | SAMN08660767 | PXGJ00000000 | SRR6951356 |
| VC1281m | PRJNA435641 | SAMN08660747 | PXGK00000000 | SRR6951358 |
| VC123n | PRJNA435641 | SAMN08660756 | PXGL00000000 | SRR6951331 |
| VC1056m | PRJNA435641 | SAMN08660758 | PXGM00000000 | SRR6951333 |
| VC1048m | PRJNA435641 | SAMN08660757 | PXGN00000000 | SRR6951330 |
| TW07865 | PRJNA435641 | SAMN08660736 | PXGO00000000 | SRR6951371 |
| Trh7 | PRJNA435641 | SAMN08660742 | PXGP00000000 | SRR6951369 |
| Trh46 | PRJNA435641 | SAMN08660740 | PXGQ00000000 | SRR6951339 |
| Trh42 | PRJNA435641 | SAMN08660741 | PXGR00000000 | SRR6951368 |
| Trh30 | PRJNA435641 | SAMN08660739 | PXGS00000000 | SRR6951374 |
| R2491 | PRJNA435641 | SAMN08660743 | PXGT00000000 | SRR6951370 |
| P2B1 | PRJNA435641 | SAMN08660752 | PXGU00000000 | SRR6951363 |
| P2A1 | PRJNA435641 | SAMN08660754 | PXGV00000000 | SRR6951349 |
| H12ESR03525 | PRJNA435641 | SAMN08660788 | PXGW00000000 | SRR6951348 |
| H12ESR01650 | PRJNA435641 | SAMN08660787 | PXGX00000000 | SRR6951347 |
| H12ESR01387 | PRJNA435641 | SAMN08660786 | PXGY00000000 | SRR6951346 |
| H12ESR01231 | PRJNA435641 | SAMN08660785 | PXGZ00000000 | SRR6951345 |
| FDE21 | PRJNA435641 | SAMN08660751 | PXHA00000000 | SRR6951362 |
| F5J | PRJNA435641 | SAMN08660745 | PXHB00000000 | SRR6951366 |
| F5F | PRJNA435641 | SAMN08660744 | PXHC00000000 | SRR6951378 |
| F1 | PRJNA435641 | SAMN08660753 | PXHD00000000 | SRR6951364 |
| ERL122034 | PRJNA435641 | SAMN08660773 | PXHE00000000 | SRR6951334 |
| ERL121829 | PRJNA435641 | SAMN08660772 | PXHF00000000 | SRR6951377 |
| ERL020412 | PRJNA435641 | SAMN08660737 | PXHG00000000 | SRR6951372 |
| AGR718 | PRJNA435641 | SAMN08660738 | PXHH00000000 | SRR6951373 |
| 54B | PRJNA435641 | SAMN08660771 | PXHI00000000 | SRR6951352 |
| 267P | PRJNA435641 | SAMN08660770 | PXHJ00000000 | SRR6951351 |
| 188B | PRJNA435641 | SAMN08660769 | PXHK00000000 | SRR6951354 |
| 16ER0517A | PRJNA435641 | SAMN08660777 | PXHL00000000 | SRR6951338 |
| 16ER0267A | PRJNA435641 | SAMN08660776 | PXHM00000000 | SRR6951340 |
| 15ER2679 | PRJNA435641 | SAMN08660784 | PXHN00000000 | SRR6951335 |
| 14ER2392 | PRJNA435641 | SAMN08660783 | PXHO00000000 | SRR6951341 |
| 13ER6723A | PRJNA435641 | SAMN08660774 | PXHP00000000 | SRR6951367 |
| 13ER6227 | PRJNA435641 | SAMN08660782 | PXHQ00000000 | SRR6951342 |
| 13ER5640 | PRJNA435641 | SAMN08660781 | PXHR00000000 | SRR6951343 |
| 13ER5154 | PRJNA435641 | SAMN08660780 | PXHS00000000 | SRR6951344 |
| 13ER5056 | PRJNA435641 | SAMN08660779 | PXHT00000000 | SRR6951365 |
| 13ER4824 | PRJNA435641 | SAMN08660778 | PXHU00000000 | SRR6951376 |
| 13ER3103A | PRJNA435641 | SAMN08660775 | PXHV00000000 | SRR6951375 |
| 116B | PRJNA435641 | SAMN08660768 | PXHW00000000 | SRR6951353 |
