# Supplementary material for: Genomic epidemiology and carbon metabolism of Escherichia coli serogroup O145 reflect contrasting phylogenies
Source: PLoS One. 2020 Jun 25;15(6):e0235066. doi: 10.1371/journal.pone.0235066 (PMC7316241; doi:10.1371/journal.pone.0235066)
Supplement: S4 Table — (DOCX) [file pone.0235066.s004.docx]

**Table S4: Virulence factors identified from serogroup O145 whole genome sequence data (n=122)**

| **Isolate** | ***stx1*** | | ***stx2*** | | | ***eae*** | | | ***ehxA*** | | | ***cba*** | | | ***astA*** | | | ***cif*** | | | ***efa1*** | | | ***espA*** | | | ***espB*** | | | ***espC*** | | | ***espF*** | | ***espI*** | | | ***espJ*** | | | ***espP*** | | | ***etpD*** | | | ***gad*** | | | ***iha*** | | | ***iss*** | | | ***katP*** | | | ***mchB*** | | | ***mchC*** | | | ***mchF*** | | | ***mcmA*** | | | ***nleA*** | | | ***nleB*** | | | ***nleC*** | | | ***perA*** | | | ***tccP*** | | | ***tir*** | | | ***toxB*** | | | ***tsh*** | | | ***cma*** | | | ***iroN*** | | | ***celb*** | | | ***lpfA*** | | | ***bfpA*** | | |
| --- | --- | --- | --- | --- | --- | --- | --- | --- | --- | --- | --- | --- | --- | --- | --- | --- | --- | --- | --- | --- | --- | --- | --- | --- | --- | --- | --- | --- | --- | --- | --- | --- | --- | --- | --- | --- | --- | --- | --- | --- | --- | --- | --- | --- | --- | --- | --- | --- | --- | --- | --- | --- | --- | --- | --- | --- | --- | --- | --- | --- | --- | --- | --- | --- | --- | --- | --- | --- | --- | --- | --- | --- | --- | --- | --- | --- | --- | --- | --- | --- | --- | --- | --- | --- | --- | --- | --- | --- | --- | --- | --- | --- | --- | --- | --- | --- | --- | --- | --- | --- | --- | --- | --- | --- | --- | --- | --- | --- | --- |
| 130322 | - | | + | | | - | | | + | | | - | | | + | | | - | | | - | | | + | | | + | | | - | | | + | | + | | | + | | | + | | | - | | | + | | | + | | | + | | | + | | | - | | | - | | | - | | | - | | | + | | | + | | | + | | | - | | | + | | | + | | | + | | | - | | | - | | | - | | | - | | | - | | | - | | |
| 132030 | - | | + | | | + | | | + | | | - | | | + | | | + | | | - | | | + | | | + | | | - | | | - | | + | | | + | | | + | | | - | | | + | | | + | | | + | | | + | | | - | | | - | | | - | | | - | | | + | | | + | | | + | | | - | | | - | | | + | | | + | | | - | | | - | | | - | | | - | | | - | | | - | | |
| 170303 | - | | - | | | + | | | - | | | - | | | + | | | - | | | - | | | + | | | - | | | + | | | + | | - | | | - | | | - | | | - | | | + | | | - | | | - | | | - | | | - | | | - | | | - | | | - | | | - | | | + | | | - | | | - | | | - | | | + | | | - | | | - | | | - | | | - | | | - | | | - | | | + | | |
| 173582 | - | | + | | | + | | | + | | | - | | | + | | | + | | | - | | | + | | | + | | | - | | | + | | - | | | + | | | + | | | - | | | + | | | + | | | + | | | - | | | - | | | - | | | - | | | - | | | + | | | + | | | + | | | - | | | + | | | + | | | + | | | - | | | - | | | - | | | - | | | - | | | - | | |
| 173758 | + | | - | | | + | | | + | | | - | | | + | | | + | | | - | | | + | | | + | | | - | | | + | | - | | | + | | | + | | | - | | | + | | | + | | | + | | | + | | | - | | | - | | | - | | | - | | | + | | | + | | | + | | | - | | | + | | | + | | | - | | | - | | | - | | | - | | | - | | | - | | | - | | |
| 182131 | - | | - | | | + | | | - | | | - | | | - | | | + | | | - | | | + | | | + | | | - | | | + | | - | | | - | | | - | | | - | | | + | | | - | | | + | | | - | | | - | | | - | | | - | | | - | | | - | | | + | | | - | | | - | | | - | | | + | | | - | | | - | | | - | | | - | | | - | | | - | | | - | | |
| 199816 | - | | - | | | + | | | - | | | - | | | - | | | + | | | - | | | + | | | - | | | + | | | + | | - | | | - | | | - | | | - | | | + | | | - | | | - | | | - | | | - | | | - | | | - | | | - | | | - | | | - | | | - | | | - | | | - | | | + | | | - | | | - | | | - | | | - | | | - | | | - | | | - | | |
| 201499 | - | | - | | | + | | | - | | | - | | | - | | | + | | | - | | | + | | | - | | | + | | | + | | - | | | - | | | - | | | - | | | + | | | - | | | - | | | - | | | - | | | - | | | - | | | - | | | - | | | - | | | - | | | - | | | - | | | + | | | - | | | - | | | - | | | - | | | - | | | - | | | - | | |
| 203972 | - | | - | | | + | | | - | | | - | | | + | | | - | | | - | | | + | | | - | | | + | | | + | | - | | | - | | | - | | | - | | | + | | | - | | | - | | | - | | | - | | | - | | | - | | | - | | | - | | | + | | | - | | | - | | | - | | | + | | | + | | | - | | | - | | | - | | | - | | | - | | | + | | |
| 241761 | - | | + | | | + | | | + | | | - | | | + | | | + | | | - | | | + | | | + | | | - | | | - | | + | | | + | | | + | | | - | | | + | | | + | | | + | | | + | | | - | | | - | | | - | | | - | | | + | | | + | | | + | | | - | | | - | | | + | | | + | | | - | | | - | | | - | | | - | | | - | | | - | | |
| 73858 | - | | + | | | + | | | + | | | - | | | - | | | - | | | + | | | + | | | + | | | - | | | + | | + | | | + | | | - | | | - | | | + | | | - | | | + | | | - | | | - | | | - | | | - | | | - | | | + | | | + | | | - | | | - | | | - | | | + | | | - | | | + | | | - | | | - | | | - | | | + | | | - | | |
| TW07865 | - | | + | | | + | | | + | | | - | | | + | | | + | | | + | | | + | | | + | | | - | | | - | | - | | | + | | | - | | | + | | | + | | | + | | | - | | | - | | | - | | | - | | | - | | | - | | | + | | | + | | | + | | | - | | | - | | | + | | | - | | | - | | | - | | | - | | | - | | | - | | | - | | |
| 116B | - | | - | | | + | | | + | | | + | | | - | | | + | | | + | | | + | | | + | | | - | | | + | | + | | | + | | | - | | | + | | | + | | | - | | | + | | | - | | | - | | | - | | | - | | | - | | | + | | | + | | | + | | | - | | | - | | | + | | | - | | | - | | | - | | | - | | | - | | | - | | | - | | |
| 13ER3103A | - | | + | | | + | | | + | | | - | | | + | | | + | | | - | | | + | | | + | | | - | | | - | | - | | | + | | | + | | | - | | | + | | | + | | | + | | | + | | | - | | | - | | | - | | | - | | | + | | | + | | | + | | | - | | | - | | | + | | | + | | | - | | | - | | | - | | | - | | | - | | | - | | |
| 13ER4824 | - | | + | | | + | | | + | | | - | | | + | | | + | | | - | | | + | | | + | | | - | | | + | | + | | | + | | | + | | | - | | | + | | | + | | | + | | | + | | | - | | | - | | | - | | | - | | | + | | | + | | | + | | | - | | | - | | | + | | | + | | | - | | | - | | | - | | | - | | | - | | | - | | |
| 13ER5056 | - | | | + | | | + | | | + | | | - | | | + | | | + | | | - | | | + | | | + | | | - | | | - | | | + | | | + | | | + | | | - | | | + | | | + | | | + | | | + | | | - | | | - | | | - | | | - | | | + | | | + | | | + | | | - | | | - | | | + | | | + | | | - | | | - | | | - | | | - | | | - | | | - |
| 13ER5154 | - | | | + | | | + | | | + | | | - | | | + | | | + | | | - | | | + | | | + | | | - | | | + | | | + | | | + | | | + | | | - | | | + | | | + | | | + | | | + | | | - | | | - | | | - | | | - | | | + | | | + | | | + | | | - | | | - | | | + | | | + | | | - | | | - | | | - | | | - | | | - | | | - |
| 13ER5640 | - | + | | | + | | | + | | | - | | | + | | | + | | | - | | | + | | | + | | | - | | | + | | | | + | | | + | | | + | | | - | | | + | | | + | | | + | | | + | | | - | | | - | | | - | | | - | | | + | | | + | | | + | | | - | | | + | | | + | | | + | | | - | | | - | | | - | | | - | | | - | | | - | |
| 13ER6227 | - | - | | | + | | | + | | | - | | | + | | | + | | | - | | | + | | | + | | | - | | | - | | | | + | | | + | | | + | | | - | | | + | | | + | | | + | | | + | | | - | | | - | | | - | | | - | | | + | | | + | | | + | | | - | | | + | | | + | | | + | | | - | | | - | | | - | | | - | | | - | | | - | |
| 13ER6723A | - | + | | | + | | | - | | | - | | | + | | | - | | | - | | | + | | | - | | | + | | | + | | | | - | | | - | | | - | | | - | | | + | | | - | | | - | | | - | | | - | | | - | | | - | | | - | | | - | | | - | | | - | | | - | | | - | | | + | | | - | | | - | | | - | | | - | | | - | | | - | | | - | |
| 143974 | - | + | | | + | | | - | | | - | | | + | | | - | | | - | | | + | | | - | | | + | | | + | | | | - | | | - | | | - | | | - | | | - | | | - | | | - | | | - | | | - | | | - | | | - | | | - | | | - | | | + | | | - | | | - | | | - | | | + | | | - | | | - | | | - | | | - | | | - | | | - | | | + | |
| 14ER2392 | - | + | | | + | | | + | | | - | | | + | | | + | | | - | | | + | | | + | | | - | | | - | | | | + | | | + | | | + | | | - | | | + | | | + | | | + | | | + | | | - | | | - | | | - | | | - | | | + | | | + | | | + | | | - | | | - | | | + | | | + | | | - | | | - | | | - | | | - | | | - | | | - | |
| 15ER2679 | - | - | | | + | | | + | | | - | | | + | | | + | | | - | | | + | | | + | | | - | | | + | | | | + | | | + | | | + | | | - | | | + | | | + | | | + | | | + | | | - | | | - | | | - | | | - | | | + | | | + | | | + | | | - | | | + | | | + | | | + | | | - | | | - | | | - | | | - | | | - | | | - | |
| 16ER0267A | + | - | | | + | | | + | | | + | | | - | | | + | | | - | | | + | | | + | | | - | | | + | | | | + | | | + | | | - | | | + | | | + | | | - | | | + | | | - | | | - | | | - | | | - | | | - | | | + | | | + | | | + | | | - | | | + | | | + | | | - | | | - | | | - | | | - | | | - | | | - | | | - | |
| 16ER0517A | + | - | | | + | | | + | | | + | | | - | | | + | | | + | | | + | | | + | | | - | | | + | | | | + | | | + | | | - | | | + | | | + | | | - | | | + | | | - | | | - | | | - | | | - | | | - | | | + | | | + | | | + | | | - | | | + | | | + | | | - | | | - | | | - | | | - | | | - | | | - | | | - | |
| 188B | - | - | | | + | | | + | | | - | | | - | | | + | | | + | | | + | | | + | | | - | | | + | | | | + | | | + | | | - | | | + | | | + | | | - | | | + | | | - | | | - | | | - | | | - | | | - | | | + | | | + | | | + | | | - | | | + | | | + | | | - | | | - | | | - | | | - | | | - | | | - | | | - | |
| 2009C3292 | - | + | | | + | | | + | | | + | | | + | | | - | | | + | | | + | | | + | | | - | | | + | | | | + | | | + | | | - | | | - | | | + | | | - | | | + | | | - | | | - | | | - | | | - | | | - | | | + | | | + | | | + | | | - | | | + | | | + | | | - | | | + | | | - | | | - | | | - | | | - | | | - | |
| 2010C3507 | - | + | | | + | | | + | | | - | | | + | | | - | | | - | | | + | | | + | | | - | | | - | | | | + | | | + | | | + | | | - | | | + | | | + | | | + | | | - | | | - | | | - | | | - | | | - | | | + | | | + | | | + | | | - | | | + | | | + | | | + | | | - | | | - | | | - | | | - | | | - | | | - | |
| 2010C3508 | - | + | | | + | | | + | | | - | | | + | | | + | | | - | | | + | | | + | | | - | | | - | | | | + | | | + | | | + | | | - | | | + | | | + | | | + | | | - | | | - | | | - | | | - | | | - | | | + | | | + | | | + | | | - | | | + | | | + | | | + | | | - | | | - | | | - | | | - | | | - | | | - | |
| 2010C3509 | - | + | | | + | | | + | | | - | | | + | | | + | | | - | | | + | | | + | | | - | | | + | | | | + | | | + | | | + | | | - | | | + | | | + | | | + | | | - | | | - | | | - | | | - | | | - | | | + | | | + | | | + | | | - | | | + | | | + | | | + | | | - | | | - | | | - | | | - | | | - | | | - | |
| 2010C3510 | - | + | | | + | | | + | | | - | | | + | | | + | | | - | | | + | | | + | | | - | | | + | | | | + | | | + | | | + | | | - | | | + | | | + | | | + | | | - | | | - | | | - | | | - | | | - | | | + | | | + | | | + | | | - | | | + | | | + | | | + | | | - | | | - | | | - | | | - | | | - | | | - | |
| 2010C3526 | - | + | | | + | | | + | | | - | | | + | | | + | | | - | | | + | | | + | | | - | | | + | | | | + | | | + | | | + | | | - | | | + | | | + | | | + | | | - | | | - | | | - | | | - | | | - | | | + | | | + | | | + | | | - | | | + | | | + | | | + | | | - | | | - | | | - | | | - | | | - | | | - | |
| 2012C4474 | - | + | | | + | | | + | | | - | | | + | | | + | | | - | | | + | | | + | | | - | | | + | | | | - | | | + | | | - | | | - | | | + | | | + | | | + | | | - | | | - | | | - | | | - | | | - | | | + | | | + | | | + | | | - | | | + | | | + | | | - | | | - | | | - | | | - | | | - | | | - | | | - | |
| 2012C4477 | - | + | | | + | | | - | | | - | | | + | | | + | | | - | | | + | | | + | | | - | | | - | | | | - | | | + | | | - | | | - | | | + | | | + | | | + | | | - | | | - | | | - | | | - | | | - | | | + | | | + | | | + | | | - | | | + | | | + | | | - | | | - | | | - | | | - | | | - | | | - | | | - | |
| 2012C4478 | - | + | | | + | | | - | | | - | | | + | | | + | | | - | | | + | | | + | | | - | | | + | | | | - | | | + | | | - | | | - | | | + | | | + | | | + | | | - | | | - | | | - | | | - | | | - | | | + | | | + | | | + | | | - | | | + | | | + | | | - | | | - | | | - | | | - | | | - | | | - | | | - | |
| 2012C4479 | - | + | | | + | | | - | | | - | | | + | | | + | | | - | | | + | | | + | | | - | | | + | | | | - | | | + | | | - | | | - | | | + | | | + | | | + | | | - | | | - | | | - | | | - | | | - | | | + | | | + | | | + | | | - | | | - | | | + | | | - | | | - | | | - | | | - | | | - | | | - | | | - | |
| 238454 | - | - | | | + | | | + | | | - | | | + | | | + | | | - | | | + | | | + | | | - | | | - | | | | - | | | + | | | + | | | - | | | + | | | + | | | + | | | + | | | - | | | - | | | - | | | - | | | + | | | + | | | + | | | - | | | + | | | + | | | - | | | - | | | - | | | - | | | - | | | - | | | - | |
| 241810 | - | - | | | + | | | + | | | - | | | + | | | + | | | - | | | + | | | + | | | - | | | + | | | | - | | | + | | | + | | | - | | | + | | | + | | | + | | | + | | | - | | | - | | | - | | | - | | | + | | | + | | | + | | | - | | | + | | | + | | | + | | | - | | | - | | | - | | | - | | | - | | | - | |
| 267P | - | - | | | + | | | + | | | + | | | - | | | + | | | + | | | + | | | + | | | - | | | + | | | | - | | | + | | | - | | | + | | | + | | | - | | | + | | | - | | | - | | | - | | | - | | | - | | | + | | | + | | | + | | | - | | | + | | | + | | | - | | | - | | | - | | | - | | | - | | | - | | | - | |
| 54B | - | - | | | + | | | + | | | + | | | - | | | + | | | + | | | + | | | + | | | - | | | + | | | | - | | | + | | | - | | | + | | | + | | | - | | | + | | | - | | | - | | | - | | | - | | | - | | | + | | | + | | | + | | | - | | | + | | | + | | | - | | | - | | | - | | | - | | | - | | | - | | | - | |
| 82EZXG | - | + | | | + | | | + | | | - | | | + | | | + | | | - | | | + | | | + | | | - | | | - | | | | + | | | + | | | + | | | - | | | + | | | + | | | + | | | + | | | - | | | - | | | - | | | - | | | + | | | + | | | + | | | - | | | - | | | + | | | + | | | - | | | - | | | - | | | - | | | - | | | - | |
| AA053 | - | - | | | + | | | + | | | - | | | + | | | + | | | - | | | + | | | + | | | - | | | + | | | | - | | | + | | | - | | | + | | | + | | | + | | | + | | | + | | | - | | | - | | | - | | | - | | | + | | | + | | | + | | | + | | | + | | | + | | | + | | | - | | | - | | | - | | | - | | | - | | | - | |
| Agr718 | - | - | | | + | | | + | | | - | | | + | | | + | | | + | | | + | | | + | | | - | | | - | | | | - | | | + | | | - | | | + | | | + | | | + | | | - | | | - | | | - | | | - | | | - | | | - | | | + | | | + | | | + | | | - | | | - | | | + | | | - | | | - | | | - | | | - | | | - | | | - | | | - | |
| BCW4180 | - | - | | | + | | | + | | | - | | | + | | | + | | | + | | | + | | | + | | | - | | | - | | | | - | | | + | | | - | | | + | | | + | | | + | | | + | | | + | | | - | | | - | | | - | | | - | | | + | | | + | | | + | | | - | | | + | | | + | | | - | | | - | | | - | | | - | | | - | | | - | | | - | |
| BYSO3C | - | + | | | + | | | - | | | - | | | + | | | + | | | - | | | + | | | - | | | + | | | + | | | | - | | | - | | | - | | | - | | | + | | | - | | | - | | | - | | | - | | | - | | | - | | | - | | | - | | | + | | | - | | | - | | | - | | | + | | | - | | | - | | | - | | | - | | | - | | | - | | | + | |
| ED657 | - | + | | | + | | | + | | | + | | | + | | | + | | | - | | | + | | | + | | | - | | | - | | | | - | | | + | | | + | | | - | | | + | | | + | | | + | | | - | | | - | | | - | | | - | | | - | | | + | | | + | | | + | | | - | | | + | | | + | | | + | | | - | | | - | | | - | | | - | | | - | | | - | |
| ERL120412 | - | - | | | + | | | + | | | - | | | - | | | - | | | + | | | + | | | + | | | - | | | + | | | | - | | | - | | | - | | | + | | | + | | | + | | | - | | | - | | | - | | | - | | | - | | | - | | | + | | | + | | | + | | | - | | | - | | | + | | | - | | | - | | | - | | | - | | | - | | | - | | | - | |
| ERL121829 | - | - | | | + | | | + | | | - | | | + | | | + | | | - | | | + | | | + | | | - | | | - | | | | + | | | + | | | + | | | - | | | + | | | + | | | + | | | + | | | + | | | + | | | + | | | + | | | + | | | + | | | + | | | - | | | - | | | + | | | + | | | - | | | - | | | - | | | - | | | - | | | - | |
| ERL122034 | - | - | | | + | | | + | | | + | | | - | | | + | | | + | | | + | | | + | | | - | | | + | | | | + | | | + | | | - | | | + | | | + | | | - | | | + | | | - | | | - | | | - | | | - | | | - | | | + | | | + | | | + | | | - | | | - | | | + | | | - | | | - | | | - | | | - | | | - | | | - | | | - | |
| F1 | - | - | | | + | | | + | | | - | | | + | | | + | | | - | | | + | | | + | | | - | | | - | | | | + | | | + | | | + | | | - | | | + | | | + | | | + | | | + | | | - | | | - | | | - | | | - | | | + | | | + | | | + | | | - | | | + | | | + | | | + | | | - | | | - | | | - | | | - | | | - | | | - | |
| F5F | - | - | | | + | | | + | | | - | | | + | | | + | | | - | | | + | | | + | | | - | | | + | | | | + | | | + | | | + | | | - | | | + | | | + | | | + | | | + | | | - | | | - | | | - | | | - | | | + | | | + | | | + | | | - | | | - | | | + | | | + | | | - | | | - | | | - | | | - | | | - | | | - | |
| F5J | - | - | | | + | | | + | | | - | | | + | | | + | | | - | | | + | | | + | | | - | | | - | | | | + | | | + | | | + | | | - | | | + | | | + | | | + | | | + | | | - | | | - | | | - | | | - | | | + | | | + | | | + | | | - | | | - | | | + | | | + | | | - | | | - | | | - | | | - | | | - | | | - | |
| FDE21 | - | - | | | + | | | + | | | - | | | + | | | + | | | - | | | + | | | + | | | + | | | - | | | | + | | | + | | | + | | | - | | | + | | | + | | | + | | | + | | | - | | | - | | | - | | | - | | | + | | | + | | | + | | | - | | | - | | | + | | | + | | | - | | | - | | | - | | | - | | | - | | | - | |
| FSIS1400369 | + | - | | | + | | | + | | | - | | | + | | | + | | | - | | | + | | | + | | | - | | | - | | | | - | | | + | | | + | | | - | | | + | | | + | | | + | | | - | | | - | | | - | | | - | | | - | | | + | | | + | | | + | | | - | | | - | | | + | | | + | | | - | | | - | | | - | | | - | | | - | | | - | |
| FSIS1500788 | - | - | | | + | | | + | | | - | | | + | | | + | | | - | | | + | | | + | | | - | | | + | | | | - | | | + | | | + | | | - | | | + | | | + | | | + | | | + | | | - | | | - | | | - | | | - | | | + | | | + | | | + | | | - | | | + | | | + | | | - | | | - | | | - | | | - | | | - | | | - | | | - | |
| FSIS1500875 | + | - | | | + | | | + | | | - | | | + | | | + | | | - | | | + | | | + | | | - | | | + | | | | - | | | + | | | + | | | - | | | + | | | + | | | + | | | + | | | - | | | - | | | - | | | - | | | + | | | + | | | + | | | - | | | - | | | + | | | - | | | - | | | - | | | - | | | - | | | - | | | - | |
| FSIS1501198 | + | - | | | + | | | + | | | - | | | + | | | + | | | - | | | + | | | + | | | - | | | + | | | | - | | | + | | | + | | | - | | | + | | | + | | | + | | | + | | | - | | | - | | | - | | | - | | | + | | | + | | | + | | | - | | | + | | | + | | | + | | | - | | | - | | | - | | | - | | | - | | | - | |
| FSIS1501717 | + | + | | | + | | | + | | | - | | | + | | | + | | | - | | | + | | | + | | | - | | | + | | | | - | | | + | | | + | | | - | | | + | | | + | | | + | | | + | | | - | | | - | | | - | | | - | | | + | | | + | | | + | | | - | | | + | | | + | | | + | | | - | | | - | | | - | | | - | | | - | | | - | |
| FSIS1502535 | + | + | | | + | | | + | | | - | | | + | | | + | | | - | | | + | | | + | | | - | | | + | | | | - | | | + | | | + | | | - | | | + | | | + | | | + | | | + | | | - | | | - | | | - | | | - | | | + | | | + | | | + | | | - | | | - | | | + | | | - | | | - | | | - | | | - | | | - | | | - | | | - | |
| FSIS1502550 | + | + | | | + | | | + | | | - | | | + | | | + | | | - | | | + | | | + | | | - | | | + | | | | - | | | + | | | + | | | - | | | + | | | + | | | + | | | + | | | - | | | - | | | - | | | - | | | + | | | + | | | + | | | - | | | + | | | + | | | + | | | - | | | - | | | - | | | - | | | - | | | - | |
| FSIS1502554 | + | + | | | + | | | + | | | - | | | + | | | + | | | - | | | + | | | + | | | - | | | - | | | | - | | | + | | | + | | | - | | | + | | | + | | | + | | | + | | | - | | | - | | | - | | | - | | | + | | | + | | | + | | | - | | | + | | | + | | | + | | | - | | | - | | | - | | | - | | | - | | | - | |
| FSIS1502976 | + | + | | | + | | | - | | | - | | | + | | | + | | | - | | | + | | | + | | | - | | | - | | | | - | | | + | | | - | | | - | | | + | | | + | | | + | | | - | | | - | | | - | | | - | | | - | | | + | | | + | | | + | | | - | | | + | | | + | | | - | | | - | | | - | | | - | | | - | | | - | | | - | |
| FSIS1502978 | + | + | | | + | | | + | | | - | | | + | | | + | | | - | | | + | | | + | | | - | | | + | | | | - | | | + | | | + | | | - | | | + | | | + | | | + | | | + | | | - | | | - | | | - | | | - | | | + | | | + | | | + | | | - | | | + | | | + | | | - | | | - | | | - | | | - | | | - | | | - | | | - | |
| FSIS1503305 | + | - | | | + | | | + | | | - | | | + | | | + | | | - | | | + | | | + | | | - | | | + | | | | - | | | + | | | + | | | - | | | + | | | + | | | + | | | + | | | - | | | - | | | - | | | - | | | + | | | + | | | + | | | - | | | + | | | + | | | - | | | - | | | - | | | - | | | - | | | - | | | - | |
| FSIS1503307 | - | + | | | + | | | + | | | - | | | + | | | + | | | - | | | + | | | + | | | - | | | + | | | | - | | | + | | | + | | | - | | | + | | | + | | | + | | | + | | | - | | | - | | | - | | | - | | | + | | | + | | | + | | | - | | | + | | | + | | | - | | | - | | | - | | | - | | | - | | | - | | | - | |
| FSIS1504619 | + | - | | | + | | | + | | | - | | | + | | | + | | | - | | | + | | | + | | | - | | | + | | | | - | | | + | | | + | | | - | | | + | | | + | | | + | | | + | | | - | | | - | | | - | | | - | | | + | | | + | | | + | | | - | | | - | | | + | | | + | | | - | | | - | | | - | | | - | | | - | | | - | |
| FSIS1505314 | - | + | | | + | | | + | | | - | | | + | | | + | | | - | | | + | | | + | | | - | | | - | | | | - | | | + | | | + | | | - | | | + | | | + | | | + | | | + | | | - | | | - | | | - | | | - | | | + | | | + | | | + | | | - | | | - | | | + | | | + | | | - | | | - | | | - | | | - | | | - | | | - | |
| FSIS1605419 | - | + | | | + | | | + | | | - | | | + | | | + | | | - | | | + | | | + | | | - | | | + | | | | + | | | + | | | + | | | - | | | + | | | + | | | + | | | + | | | - | | | - | | | - | | | - | | | + | | | + | | | + | | | - | | | + | | | + | | | + | | | - | | | - | | | - | | | - | | | - | | | - | |
| FSIS1605420 | - | + | | | + | | | + | | | - | | | + | | | + | | | - | | | + | | | + | | | - | | | + | | | | + | | | + | | | + | | | - | | | + | | | + | | | + | | | + | | | - | | | - | | | - | | | - | | | + | | | + | | | + | | | - | | | - | | | + | | | + | | | - | | | - | | | - | | | - | | | - | | | - | |
| FSIS1605733 | + | + | | | + | | | + | | | - | | | + | | | + | | | - | | | + | | | + | | | - | | | + | | | | - | | | + | | | + | | | - | | | + | | | + | | | + | | | + | | | - | | | - | | | - | | | - | | | + | | | + | | | + | | | - | | | + | | | + | | | - | | | - | | | - | | | - | | | - | | | - | | | - | |
| FSIS1700607 | - | + | | | + | | | + | | | - | | | + | | | + | | | - | | | + | | | + | | | - | | | - | | | | + | | | + | | | + | | | - | | | + | | | + | | | + | | | + | | | - | | | - | | | - | | | - | | | + | | | + | | | + | | | - | | | - | | | + | | | + | | | - | | | - | | | - | | | - | | | - | | | - | |
| H12ESR01231 | - | - | | | + | | | + | | | - | | | + | | | + | | | - | | | + | | | + | | | - | | | + | | | | + | | | + | | | + | | | - | | | + | | | + | | | + | | | + | | | - | | | - | | | - | | | - | | | + | | | + | | | + | | | - | | | + | | | + | | | + | | | - | | | - | | | - | | | - | | | - | | | - | |
| H12ESR01387 | - | + | | | + | | | + | | | - | | | + | | | + | | | - | | | + | | | + | | | - | | | - | | | | + | | | + | | | + | | | - | | | + | | | + | | | + | | | + | | | - | | | - | | | - | | | - | | | + | | | + | | | + | | | - | | | - | | | + | | | + | | | - | | | - | | | - | | | - | | | - | | | - | |
| H12ESR01650 | - | - | | | + | | | + | | | - | | | + | | | + | | | - | | | + | | | + | | | - | | | - | | | | + | | | + | | | + | | | - | | | + | | | + | | | + | | | + | | | - | | | - | | | - | | | - | | | + | | | + | | | + | | | - | | | + | | | + | | | + | | | - | | | - | | | - | | | - | | | - | | | - | |
| H12ESR03525 | - | + | | | + | | | + | | | - | | | + | | | + | | | - | | | + | | | + | | | - | | | - | | | | + | | | + | | | + | | | - | | | + | | | + | | | + | | | + | | | - | | | - | | | - | | | - | | | + | | | + | | | + | | | - | | | + | | | + | | | + | | | - | | | - | | | - | | | - | | | - | | | - | |
| MOD1EC1641 | + | - | | | + | | | + | | | - | | | + | | | + | | | - | | | + | | | + | | | - | | | - | | | | - | | | + | | | + | | | - | | | + | | | + | | | + | | | + | | | - | | | - | | | - | | | - | | | + | | | + | | | - | | | - | | | + | | | + | | | - | | | - | | | - | | | - | | | - | | | - | | | - | |
| MOD1EC1661 | + | - | | | + | | | + | | | - | | | + | | | + | | | - | | | + | | | + | | | - | | | - | | | | - | | | + | | | + | | | - | | | + | | | + | | | + | | | + | | | - | | | - | | | - | | | - | | | + | | | + | | | + | | | - | | | - | | | + | | | - | | | - | | | - | | | - | | | - | | | - | | | - | |
| MOD1EC1672 | - | + | | | + | | | + | | | + | | | + | | | - | | | - | | | + | | | + | | | - | | | + | | | | + | | | + | | | - | | | - | | | - | | | - | | | + | | | - | | | - | | | - | | | - | | | - | | | + | | | + | | | - | | | - | | | + | | | + | | | - | | | + | | | - | | | - | | | - | | | + | | | - | |
| MOD1EC1935 | - | + | | | + | | | + | | | - | | | + | | | + | | | - | | | + | | | + | | | - | | | - | | | | + | | | + | | | + | | | - | | | + | | | + | | | + | | | + | | | - | | | - | | | - | | | - | | | + | | | + | | | - | | | - | | | - | | | + | | | - | | | - | | | - | | | - | | | - | | | - | | | - | |
| MOD1EC1941 | - | - | | | + | | | - | | | + | | | + | | | - | | | + | | | + | | | + | | | - | | | + | | | | + | | | + | | | - | | | - | | | + | | | - | | | + | | | - | | | - | | | - | | | - | | | - | | | + | | | + | | | + | | | - | | | + | | | + | | | - | | | - | | | - | | | - | | | + | | | + | | | - | |
| MOD1EC1954 | - | + | | | + | | | + | | | - | | | + | | | + | | | - | | | + | | | + | | | - | | | - | | | | - | | | + | | | - | | | + | | | + | | | + | | | - | | | - | | | - | | | - | | | - | | | - | | | + | | | + | | | - | | | - | | | + | | | + | | | - | | | - | | | - | | | - | | | - | | | - | | | - | |
| MOD1EC1969 | + | - | | | + | | | + | | | - | | | + | | | + | | | - | | | + | | | + | | | - | | | + | | | | - | | | + | | | + | | | - | | | + | | | + | | | + | | | - | | | - | | | - | | | - | | | - | | | + | | | + | | | + | | | - | | | - | | | + | | | + | | | - | | | - | | | - | | | - | | | - | | | - | |
| MOD1EC1971 | - | + | | | + | | | + | | | - | | | + | | | + | | | - | | | + | | | + | | | - | | | - | | | | - | | | + | | | + | | | - | | | + | | | + | | | + | | | - | | | - | | | - | | | - | | | - | | | + | | | + | | | + | | | - | | | - | | | + | | | - | | | - | | | - | | | - | | | - | | | - | | | - | |
| MOD1EC1972 | - | + | | | + | | | + | | | - | | | + | | | + | | | - | | | + | | | + | | | - | | | - | | | | + | | | + | | | + | | | - | | | + | | | + | | | + | | | - | | | - | | | - | | | - | | | - | | | + | | | + | | | + | | | - | | | - | | | + | | | + | | | - | | | - | | | - | | | - | | | - | | | - | |
| MOD1EC2002 | + | + | | | + | | | + | | | - | | | + | | | + | | | - | | | + | | | + | | | - | | | + | | | | - | | | + | | | + | | | - | | | + | | | + | | | + | | | + | | | - | | | - | | | - | | | - | | | + | | | + | | | + | | | - | | | + | | | + | | | + | | | - | | | - | | | - | | | - | | | - | | | - | |
| MOD1EC5078 | - | - | | | - | | | - | | | - | | | + | | | - | | | - | | | - | | | - | | | - | | | - | | | | - | | | - | | | - | | | - | | | + | | | - | | | - | | | - | | | - | | | - | | | - | | | - | | | - | | | - | | | - | | | - | | | - | | | - | | | - | | | - | | | - | | | - | | | - | | | - | | | - | |
| MOD1EC5081 | - | - | | | - | | | - | | | - | | | + | | | - | | | - | | | - | | | - | | | - | | | - | | | | - | | | - | | | - | | | - | | | + | | | - | | | + | | | - | | | - | | | - | | | - | | | - | | | - | | | - | | | - | | | - | | | - | | | - | | | - | | | - | | | - | | | - | | | - | | | - | | | - | |
| MOD1EC5165 | - | - | | | + | | | - | | | - | | | - | | | + | | | + | | | + | | | + | | | - | | | + | | | | - | | | + | | | - | | | - | | | + | | | - | | | + | | | - | | | - | | | - | | | + | | | - | | | + | | | + | | | - | | | - | | | - | | | + | | | - | | | - | | | + | | | + | | | - | | | - | | | - | |
| MOD1EC5842 | - | - | | | + | | | - | | | - | | | + | | | + | | | - | | | + | | | + | | | - | | | - | | | | + | | | + | | | - | | | - | | | + | | | - | | | + | | | - | | | - | | | - | | | - | | | - | | | + | | | + | | | + | | | - | | | + | | | - | | | - | | | - | | | - | | | - | | | - | | | - | | | - | |
| MOD1EC5961 | - | - | | | + | | | - | | | - | | | - | | | + | | | + | | | + | | | + | | | - | | | + | | | | - | | | + | | | - | | | - | | | - | | | - | | | + | | | - | | | - | | | - | | | + | | | - | | | + | | | + | | | - | | | - | | | - | | | + | | | - | | | - | | | + | | | + | | | - | | | - | | | - | |
| MOD1EC6028 | - | + | | | + | | | + | | | - | | | + | | | + | | | + | | | + | | | + | | | - | | | - | | | | - | | | + | | | - | | | + | | | + | | | + | | | - | | | - | | | - | | | - | | | - | | | - | | | + | | | + | | | + | | | - | | | + | | | + | | | - | | | - | | | - | | | - | | | - | | | - | | | - | |
| MOD1EC6710 | - | - | | | + | | | - | | | - | | | + | | | - | | | + | | | + | | | + | | | - | | | + | | | | + | | | + | | | + | | | - | | | - | | | - | | | + | | | - | | | - | | | - | | | - | | | - | | | + | | | + | | | + | | | - | | | + | | | + | | | - | | | - | | | - | | | - | | | + | | | - | | | - | |
| OLC0719 | + | - | | | + | | | + | | | - | | | + | | | + | | | - | | | + | | | + | | | - | | | - | | | | + | | | + | | | + | | | - | | | + | | | + | | | + | | | + | | | - | | | - | | | - | | | - | | | + | | | + | | | + | | | - | | | + | | | + | | | + | | | - | | | - | | | - | | | - | | | - | | | - | |
| OLC1258 | - | + | | | + | | | - | | | - | | | - | | | - | | | - | | | + | | | - | | | + | | | + | | | | - | | | - | | | - | | | - | | | + | | | - | | | - | | | - | | | - | | | - | | | - | | | - | | | - | | | + | | | - | | | - | | | - | | | - | | | + | | | - | | | - | | | - | | | - | | | - | | | - | |
| P2A1 | - | - | | | + | | | + | | | - | | | + | | | + | | | - | | | + | | | + | | | - | | | - | | | | + | | | + | | | + | | | - | | | + | | | + | | | + | | | + | | | - | | | - | | | - | | | - | | | + | | | + | | | + | | | - | | | + | | | + | | | + | | | - | | | - | | | - | | | - | | | - | | | - | |
| P2B1 | - | - | | | + | | | + | | | - | | | + | | | + | | | - | | | + | | | + | | | - | | | - | | | | + | | | + | | | + | | | - | | | - | | | + | | | + | | | + | | | - | | | - | | | - | | | - | | | + | | | + | | | + | | | - | | | - | | | + | | | + | | | - | | | - | | | - | | | - | | | - | | | - | |
| PNUSAE000756 | - | + | | | + | | | + | | | - | | | + | | | + | | | - | | | + | | | + | | | - | | | + | | | | + | | | + | | | + | | | - | | | + | | | + | | | + | | | + | | | - | | | - | | | - | | | - | | | + | | | + | | | + | | | - | | | + | | | + | | | + | | | - | | | - | | | - | | | - | | | - | | | - | |
| PNUSAE001244 | - | + | | | + | | | + | | | - | | | + | | | + | | | - | | | + | | | + | | | - | | | - | | | | + | | | + | | | - | | | - | | | + | | | + | | | + | | | + | | | - | | | - | | | - | | | - | | | + | | | + | | | + | | | - | | | + | | | + | | | - | | | - | | | - | | | - | | | - | | | - | | | - | |
| PNUSAE003232 | - | + | | | + | | | + | | | - | | | + | | | + | | | - | | | + | | | + | | | - | | | + | | | | - | | | + | | | + | | | - | | | + | | | + | | | + | | | - | | | - | | | - | | | - | | | - | | | + | | | + | | | + | | | - | | | + | | | + | | | + | | | - | | | - | | | - | | | - | | | - | | | - | |
| R249-1 | - | - | | | + | | | - | | | - | | | + | | | - | | | - | | | + | | | - | | | + | | | + | | | | - | | | - | | | - | | | - | | | + | | | - | | | - | | | - | | | - | | | - | | | - | | | - | | | - | | | + | | | - | | | - | | | - | | | + | | | - | | | - | | | - | | | - | | | - | | | - | | | + | |
| Trh30 | - | - | | | + | | | + | | | - | | | + | | | + | | | - | | | + | | | + | | | - | | | - | | | | - | | | + | | | - | | | - | | | + | | | + | | | + | | | + | | | - | | | - | | | - | | | - | | | + | | | + | | | + | | | - | | | + | | | + | | | + | | | - | | | - | | | - | | | - | | | - | | | - | |
| Trh42 | - | - | | | + | | | - | | | - | | | - | | | - | | | - | | | + | | | - | | | + | | | + | | | | - | | | - | | | - | | | - | | | + | | | - | | | + | | | - | | | - | | | - | | | - | | | - | | | - | | | + | | | + | | | - | | | - | | | + | | | - | | | - | | | - | | | - | | | - | | | - | | | - | |
| Trh46 | - | - | | | + | | | - | | | - | | | - | | | - | | | - | | | + | | | - | | | + | | | + | | | | - | | | - | | | - | | | - | | | + | | | - | | | - | | | - | | | - | | | - | | | - | | | - | | | - | | | + | | | + | | | - | | | - | | | + | | | - | | | - | | | - | | | - | | | - | | | - | | | - | |
| Trh7 | - | - | | | + | | | - | | | - | | | - | | | + | | | - | | | + | | | + | | | - | | | - | | | | - | | | - | | | - | | | - | | | + | | | - | | | + | | | - | | | - | | | - | | | - | | | - | | | - | | | + | | | - | | | - | | | - | | | + | | | - | | | - | | | - | | | - | | | - | | | - | | | - | |
| VC1048m | - | - | | | + | | | + | | | - | | | + | | | + | | | - | | | + | | | + | | | - | | | - | | | | - | | | + | | | - | | | + | | | + | | | + | | | - | | | - | | | - | | | - | | | - | | | - | | | + | | | + | | | + | | | - | | | + | | | + | | | - | | | - | | | - | | | - | | | - | | | - | | | - | |
| VC1056m | - | - | | | + | | | + | | | - | | | + | | | + | | | - | | | + | | | + | | | - | | | - | | | | + | | | + | | | + | | | - | | | + | | | + | | | + | | | + | | | - | | | - | | | - | | | - | | | + | | | + | | | + | | | - | | | + | | | + | | | + | | | - | | | - | | | - | | | - | | | - | | | - | |
| VC123n | - | - | | | + | | | + | | | - | | | + | | | + | | | - | | | + | | | + | | | - | | | - | | | | + | | | + | | | + | | | - | | | + | | | + | | | + | | | + | | | - | | | - | | | - | | | - | | | + | | | + | | | + | | | - | | | - | | | + | | | + | | | - | | | - | | | - | | | - | | | - | | | - | |
| VC1281m | - | - | | | + | | | + | | | - | | | + | | | + | | | - | | | + | | | + | | | - | | | - | | | | + | | | + | | | + | | | - | | | + | | | + | | | + | | | + | | | - | | | - | | | - | | | - | | | + | | | + | | | + | | | - | | | - | | | + | | | + | | | - | | | - | | | - | | | - | | | - | | | - | |
| VC1413m | - | + | | | + | | | + | | | - | | | + | | | + | | | - | | | + | | | + | | | - | | | - | | | | + | | | + | | | + | | | - | | | + | | | + | | | + | | | + | | | - | | | - | | | - | | | - | | | + | | | + | | | + | | | - | | | + | | | + | | | + | | | - | | | - | | | - | | | - | | | - | | | - | |
| VC1506m | - | - | | | + | | | + | | | - | | | + | | | + | | | - | | | + | | | + | | | - | | | - | | | | + | | | + | | | + | | | - | | | + | | | + | | | + | | | + | | | - | | | - | | | - | | | - | | | + | | | + | | | + | | | - | | | - | | | + | | | + | | | - | | | - | | | - | | | - | | | - | | | - | |
| VC194m | - | - | | | + | | | + | | | - | | | + | | | + | | | - | | | + | | | + | | | - | | | - | | | | + | | | + | | | + | | | - | | | + | | | + | | | + | | | + | | | - | | | - | | | - | | | - | | | + | | | + | | | + | | | - | | | - | | | + | | | + | | | - | | | - | | | - | | | - | | | - | | | - | |
| VC237m | - | - | | | + | | | - | | | - | | | + | | | + | | | - | | | + | | | + | | | - | | | - | | | | + | | | + | | | - | | | - | | | + | | | + | | | + | | | - | | | - | | | - | | | - | | | - | | | + | | | + | | | + | | | - | | | + | | | + | | | - | | | - | | | - | | | - | | | - | | | - | | | - | |
| VC237o | - | - | | | + | | | - | | | - | | | + | | | + | | | - | | | + | | | + | | | - | | | + | | | | + | | | + | | | - | | | - | | | + | | | + | | | + | | | - | | | - | | | - | | | - | | | - | | | + | | | + | | | + | | | - | | | + | | | + | | | - | | | - | | | - | | | - | | | - | | | - | | | - | |
| VC308m | - | - | | | + | | | + | | | - | | | + | | | + | | | - | | | + | | | + | | | - | | | - | | | | + | | | + | | | + | | | - | | | + | | | + | | | + | | | + | | | - | | | - | | | - | | | - | | | + | | | + | | | + | | | - | | | - | | | + | | | + | | | - | | | - | | | - | | | - | | | - | | | - | |
| VC476m | - | - | | | + | | | + | | | - | | | + | | | + | | | - | | | + | | | + | | | - | | | - | | | | + | | | + | | | + | | | - | | | + | | | + | | | + | | | + | | | - | | | - | | | - | | | - | | | + | | | + | | | + | | | - | | | - | | | + | | | + | | | - | | | - | | | - | | | - | | | - | | | - | |
| VC506m | - | - | | | + | | | + | | | - | | | + | | | + | | | - | | | + | | | + | | | - | | | + | | | | + | | | + | | | + | | | - | | | + | | | + | | | + | | | + | | | - | | | - | | | - | | | - | | | + | | | + | | | + | | | - | | | - | | | + | | | + | | | - | | | - | | | - | | | - | | | - | | | - | |
| VC525m | - | - | | | + | | | + | | | - | | | + | | | + | | | - | | | + | | | + | | | - | | | - | | | | + | | | + | | | + | | | - | | | + | | | + | | | + | | | + | | | - | | | - | | | - | | | - | | | + | | | + | | | + | | | - | | | + | | | + | | | + | | | - | | | - | | | - | | | - | | | - | | | - | |
| VC554m | - | - | | | + | | | + | | | - | | | + | | | + | | | - | | | + | | | + | | | - | | | - | | | | + | | | + | | | + | | | - | | | + | | | + | | | + | | | + | | | - | | | - | | | - | | | - | | | + | | | + | | | + | | | - | | | - | | | + | | | + | | | - | | | - | | | - | | | - | | | - | | | - | |
| VC847m | - | - | | | + | | | + | | | - | | | + | | | + | | | - | | | + | | | + | | | - | | | - | | | | + | | | + | | | + | | | - | | | + | | | + | | | + | | | + | | | - | | | - | | | - | | | - | | | + | | | + | | | + | | | - | | | - | | | + | | | + | | | - | | | - | | | - | | | - | | | - | | | - | |
| VC849m | - | - | | | + | | | + | | | - | | | + | | | + | | | - | | | + | | | + | | | - | | | - | | | | + | | | + | | | + | | | - | | | + | | | + | | | + | | | + | | | - | | | - | | | - | | | - | | | + | | | + | | | + | | | - | | | + | | | + | | | + | | | - | | | - | | | - | | | - | | | - | | | - | |
| VC874o | - | - | | | + | | | + | | | - | | | + | | | + | | | - | | | + | | | + | | | - | | | - | | | | - | | | + | | | + | | | - | | | + | | | + | | | + | | | + | | | - | | | - | | | - | | | - | | | + | | | + | | | + | | | - | | | - | | | + | | | + | | | - | | | - | | | - | | | - | | | - | | | - | |
| VC880m | - | - | | | + | | | + | | | - | | | + | | | + | | | - | | | + | | | + | | | - | | | - | | | | + | | | + | | | + | | | - | | | + | | | + | | | + | | | + | | | - | | | - | | | - | | | - | | | + | | | + | | | + | | | - | | | + | | | + | | | + | | | - | | | - | | | - | | | - | | | - | | | - | |
