# Supplementary material for: Genomic epidemiology and carbon metabolism of Escherichia coli serogroup O145 reflect contrasting phylogenies
Source: PLoS One. 2020 Jun 25;15(6):e0235066. doi: 10.1371/journal.pone.0235066 (PMC7316241; doi:10.1371/journal.pone.0235066)
Supplement: S5 Table — (DOCX) [file pone.0235066.s005.docx]

**Table S5: Plasmid incompatibility factors identified from serogroup O145 whole genome sequence data (n=122)**

| **Strain** | **Plasmids schemed** |
| --- | --- |
| 073858 | IncFII (pRSB107), IncFIB (AP001918) |
| 116B | IncFIB (AP001918) |
| 130322 | IncFIB (AP001918) |
| 132030 | IncFIB (AP001918) |
| 13ER3103A | IncFIB (AP001918) |
| 13ER4824 | IncFIB (AP001918) |
| 13ER5056 | IncFIB (AP001918) |
| 13ER5154 | IncFIB (AP001918) |
| 13ER5640 | IncFIB (AP001918) |
| 13ER6227 | IncFIB (AP001918) |
| 13ER6723A | None detected |
| 143974 | IncFII, IncI1, IncFIB (AP001918) |
| 14ER2392 | IncFIB (AP001918) |
| 15ER2679 | IncFIB (AP001918) |
| 16ER0267A | IncFIB (AP001918) |
| 16ER0517A | IncFIB (AP001918) |
| 170303 | IncFII, IncFIB (AP001918) |
| 173582 | IncI1, IncFIB (AP001918) |
| 173758 | IncI1, IncFIB (AP001918) |
| 182131 | IncFII (pCoo) |
| 188B | IncFIB (AP001918) |
| 199816 | None detected |
| 2009C3292 | IncFII (pRSB107), IncFIB (AP001918) |
| 2010C-3507 | IncFIB (AP001918) |
| 2010C-3508 | IncFIB (AP001918) |
| 2010C-3509 | IncFIB (AP001918) |
| 2010C-3510 | IncFIB (AP001918) |
| 2010C-3526 | IncFIB (AP001918) |
| 2012C4474 | IncB/O/K/Z, IncFIB (AP001918) |
| 2012C4477 | IncB/O/K/Z, IncFIB (AP001918) |
| 2012C4478 | IncB/O/K/Z, IncFIB (AP001918) |
| 2012C4479 | IncB/O/K/Z, IncFIB (AP001918) |
| 201499 | None detected |
| 203972 | IncFII (pHN7A8), IncFIB (AP001918) |
| 238454 | IncFIB (AP001918) |
| 241761 | IncFIB (AP001918) |
| 241810 | IncFIB (AP001918) |
| 267P | IncFIB (AP001918) |
| 54B | IncFIB (AP001918) |
| 82EZXG | IncFIB (AP001918) |
| AA053 | IncFIB (AP001918), IncFII (pHN7A8) |
| AGR718 | IncFIB (AP001918) |
| BCW4180 | IncFII, IncI1, IncFIB (AP001918) |
| BYSO3C | IncFII, IncFIB (AP001918) |
| ED657 | IncFII, IncFIB (AP001918) |
| ERL120412 | IncFIB (AP001918) |
| ERL121829 | IncFIB (AP001918) |
| ERL122034 | IncFIB (AP001918) |
| F1 | IncFIB (AP001918) |
| F5F | IncFIB (AP001918) |
| F5J | IncFIB (AP001918) |
| FDE21 | IncFIB (AP001918) |
| FSIS1400369 | IncFIB (AP001918) |
| FSIS1500788 | IncFIB (AP001918) |
| FSIS1500875 | IncFIB (AP001918) |
| FSIS1501198 | IncFIB (AP001918) |
| FSIS1501717 | IncFIB (AP001918) |
| FSIS1502535 | IncFIB (AP001918) |
| FSIS1502550 | IncFIB (AP001918) |
| FSIS1502554 | IncFIB (AP001918) |
| FSIS1502976 | None detected |
| FSIS1502978 | IncFIB (AP001918) |
| FSIS1503305 | IncFIB (AP001918) |
| FSIS1503307 | IncFIB (AP001918) |
| FSIS1504619 | IncFIB (AP001918) |
| FSIS1505314 | IncFIB (AP001918) |
| FSIS1605419 | IncFIB (AP001918) |
| FSIS1605420 | IncFIB (AP001918) |
| FSIS1605733 | IncFIB (AP001918) |
| FSIS1700607 | IncFIB (AP001918) |
| H12ESR01231 | IncFIB (AP001918) |
| H12ESR01387 | IncFIB (AP001918) |
| H12ESR01650 | IncFIB (AP001918) |
| H12ESR03525 | IncFIB (AP001918) |
| MOD1EC1641 | IncFIB (AP001918) |
| MOD1EC1661 | IncFIB (AP001918) |
| MOD1EC1672 | IncFII (pRSB107), IncFIB (AP001918) |
| MOD1EC1935 | IncFIB (AP001918) |
| MOD1EC1941 | IncFIA, IncFIB (AP001918), IncFII (pCoo) |
| MOD1EC1954 | IncFIB (AP001918) |
| MOD1EC1969 | IncFIB (AP001918) |
| MOD1EC1971 | IncFIB (AP001918) |
| MOD1EC1972 | IncFIB (AP001918) |
| MOD1-EC2002 | IncFIB (AP001918) |
| MOD1EC5078 | IncFII (pCoo) |
| MOD1EC5081 | IncFII (pHN7A8), IncFII (pCoo) |
| MOD1EC5165 | IncFII, IncFIB (AP001918) |
| MOD1EC5842 | None detected |
| MOD1-EC5961 | IncFII (29), IncI1, IncFIB (AP001918), IncN |
| MOD1EC6028 | IncFIB (AP001918) |
| MOD1-EC6710 | IncFIA, IncFIB (AP001918), IncFII (pCoo) |
| OLC0719 | IncFIB (AP001918) |
| OLC1258 None detected | |
| P2A1 | IncFIB (AP001918) |
| P2B1 | IncFIB (AP001918) |
| PNUSAE000756 | IncFIB (AP001918) |
| PNUSAE001244 | IncFIB (AP001918) |
| PNUSAE003232 | IncFIB (AP001918) |
| R249-1 | IncFII, IncFIB (AP001918) |
| Trh30 | IncFIB (AP001918) |
| Trh42 | None detected |
| Trh46 | IncI2 |
| Trh7 | IncFIB (AP001918), IncFII (29), IncFIC (FII) |
| TW07865 | IncFIB (AP001918) |
| VC1048m | IncFIB (AP001918) |
| VC1056m | IncFIB (AP001918) |
| VC123n | IncFIB (AP001918) |
| VC1281m | IncFIB (AP001918) |
| VC1413m | IncFIB (AP001918) |
| VC1506m | IncFIB (AP001918) |
| VC194m | IncFIB (AP001918) |
| VC237m | None detected |
| VC237o | None detected |
| VC308m | IncFIB (AP001918) |
| VC476m | IncFIB (AP001918) |
| VC506m | IncFIB (AP001918) |
| VC525m | IncI1, IncFIB (AP001918) |
| VC554m | IncFIB (AP001918) |
| VC847m | IncFIB (AP001918) |
| VC849m | IncFIB (AP001918) |
| VC874o | IncFIB (AP001918) |
| VC880m | IncFIB (AP001918) |
