# Supplementary material for: Genomic epidemiology and carbon metabolism of Escherichia coli serogroup O145 reflect contrasting phylogenies
Source: PLoS One. 2020 Jun 25;15(6):e0235066. doi: 10.1371/journal.pone.0235066 (PMC7316241; doi:10.1371/journal.pone.0235066)
Supplement: S6 Table — (DOCX) [file pone.0235066.s006.docx]

## Table S6: *E. coli* tRNA integration site for the locus for enterocyte effacement (LEE) pathogenicity island

| Isolate | LEE insertion site^a^ | *eae* subtype |
| --- | --- | --- |
| Trh42 | *leu*^b^ | ι |
| 073858 | *leu*^b^ | β |
| MOD1EC1941 | *pheU* | β |
| ERL122034 | *pheV* | ε |
| Trh30 | *pheV* | γ |
| Trh7 | *pheV* | β |
| VC847m | *pheV* | γ |
| 2009C-3292 | *pheV* | β |
| 54B | *pheV* | ε |
| 116B | *pheV* | ε |
| 188B | *pheV* | ε |
| 16ER0267A | *pheV* | ε |
| 267P | *pheV* | ε |
| 16ER0517A | *pheV* | ε |
| AA053 | *selC* | γ |
| AGR718 | *selC* | γ |
| BYSO3C | *selC* | ι |
| ERL020412 | *selC* | γ |
| ERL121829 | *selC* | γ |
| F1 | *selC* | γ |
| FDE2/1 | *selC* | γ |
| FSIS1502978 | *selC* | γ |
| FSIS1503307 | *selC* | γ |
| FSIS1605733 | selC | γ |
| H12ESR01231 | *selC* | γ |
| H12ESR03525 | *selC* | γ |
| MOD1EC1641 | *selC* | γ |
| MOD1EC1661 | *selC* | γ |
| MOD1EC1935 | selC | γ |
| MOD1EC1954 | selC | γ |
| MOD1EC1971 | selC | γ |
| MOD1EC1972 | *selC* | γ |
| MOD1EC5961 | *selC* | β |
| MOD1EC6028 | selC | γ |
| OLC1258 | *selC* | ι |
| P2B1 | *selC* | γ |
| PNUSA001244 | *selC* | γ |
| R2491 | *selC* | ι |
| Trh46 | *selC* | ι |
| TW07865 | *selC* | γ |
| VC1048m | *selC* | γ |
| VC1056m | *selC* | γ |
| VC1281m | *selC* | γ |
| VC1413m | *selC* | γ |
| VC1506m | *selC* | γ |
| VC237m | *selC* | γ |
| VC237o | *selC* | γ |
| VC506m | *selC* | γ |
| VC506m | *selC* | γ |
| VC554m | *selC* | γ |
| VC874o | *selC* | γ |
| VC880m | *selC* | γ |
| 2012C-4477 | *selC* | γ |
| 2010C-3526 | *selC* | γ |
| 2010C-3510 | *selC* | γ |
| 2010C-3509 | *selC* | γ |
| 2010C-3508 | *selC* | γ |
| 2010C-3507 | *selC* | γ |
| 14ER2392 | *selC* | γ |
| 15ER2679 | *selC* | γ |
| 13ER3103A | *selC* | γ |
| 13ER5056 | *selC* | γ |
| 13ER5154 | *selC* | γ |
| 13ER5640 | *selC* | γ |
| 13ER6227 | *selC* | γ |
| 13ER6723A | *selC* | ι |
| 143974 | *selC* | γ |
| 170303 | *selC* | ι |
| 173758 | *selC* | γ |
| 199816 | *selC* | α |
| 201499 | *selC* | α |
| 203972 | *selC* | ι |
| 238454 | *selC* | γ |
| 82EZXG | - | γ |
| BCW4180 | - | γ |
| ED657 | - | γ |
| F5F | - | γ |
| F5J | - | γ |
| FSIS1400369 | - | γ |
| FSIS1500788 | - | γ |
| FSIS1500875 | - | γ |
| FSIS1501198 | - | γ |
| FSIS1501717 | - | γ |
| FSIS1502535 | - | γ |
| FSIS1502550 | - | γ |
| FSIS1502554 | - | γ |
| FSIS1502976 | - | γ |
| FSIS1503305 | - | γ |
| FSIS1504619 | - | γ |
| FSIS1505314 | - | γ |
| FSIS1605419 | - | γ |
| FSIS1605420 | - | γ |
| FSIS1700607 | - | γ |
| H12ESR01387 | - | γ |
| H12ESR01650 | - | γ |
| MOD1EC1672 | - | β |
| MOD1EC1969 | - | γ |
| MOD1EC2002 | - | γ |
| MOD1EC5165 | - | β |
| MOD1EC5842 | - | γ |
| MOD1EC6710 | - | β |
| OLC0719 | - | γ |
| P2A1 | - | γ |
| PNUSA000756 | - | γ |
| PNUSA003232 | - | γ |
| VC123n | - | γ |
| VC194m | - | γ |
| VC308m | - | γ |
| VC476m | - | γ |
| VC525m | - | γ |
| VC849m | - | γ |
| 2012C-4479 | - | γ |
| 2012C-4478 | - | γ |
| 2012C-4474 | - | γ |
| 13ER4824 | - | γ |
| 130322 | - | γ |
| 132030 | - | γ |
| 173582 | - | γ |
| 182131 | - | β |
| 241761 | - | γ |
| 241810 | - | γ |

a: -, LEE pathogenicity island integration site could not be precisely determined

b: The LEE pathogenicity island integration site could not be precisely determined, however, the LEE was located near the tRNA *leu* gene
