# Supplementary figures and images for: Genomic epidemiology and carbon metabolism of Escherichia coli serogroup O145 reflect contrasting phylogenies
Source: PLoS One. 2020 Jun 25;15(6):e0235066. doi: 10.1371/journal.pone.0235066 (PMC7316241; doi:10.1371/journal.pone.0235066)

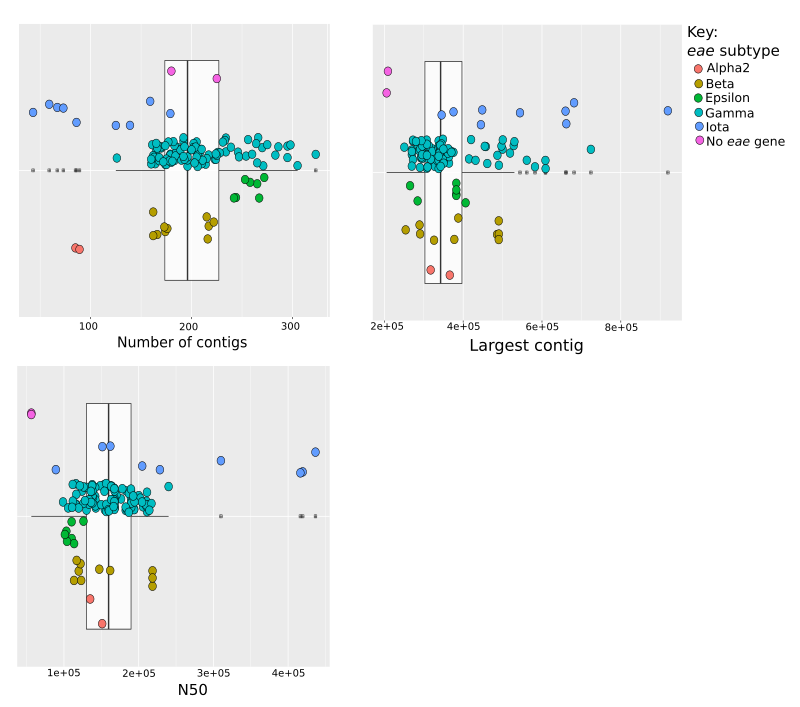

Supplement: S1 Fig — The box and whisker plots indicate the number of contigs, largest contig and N50 value for the serogroup O145 strains (n = 122). Each data point is shown on the plots and has been colour coded according to eae subtype, as indicated by the figure key. (PNG) [file pone.0235066.s007.png]

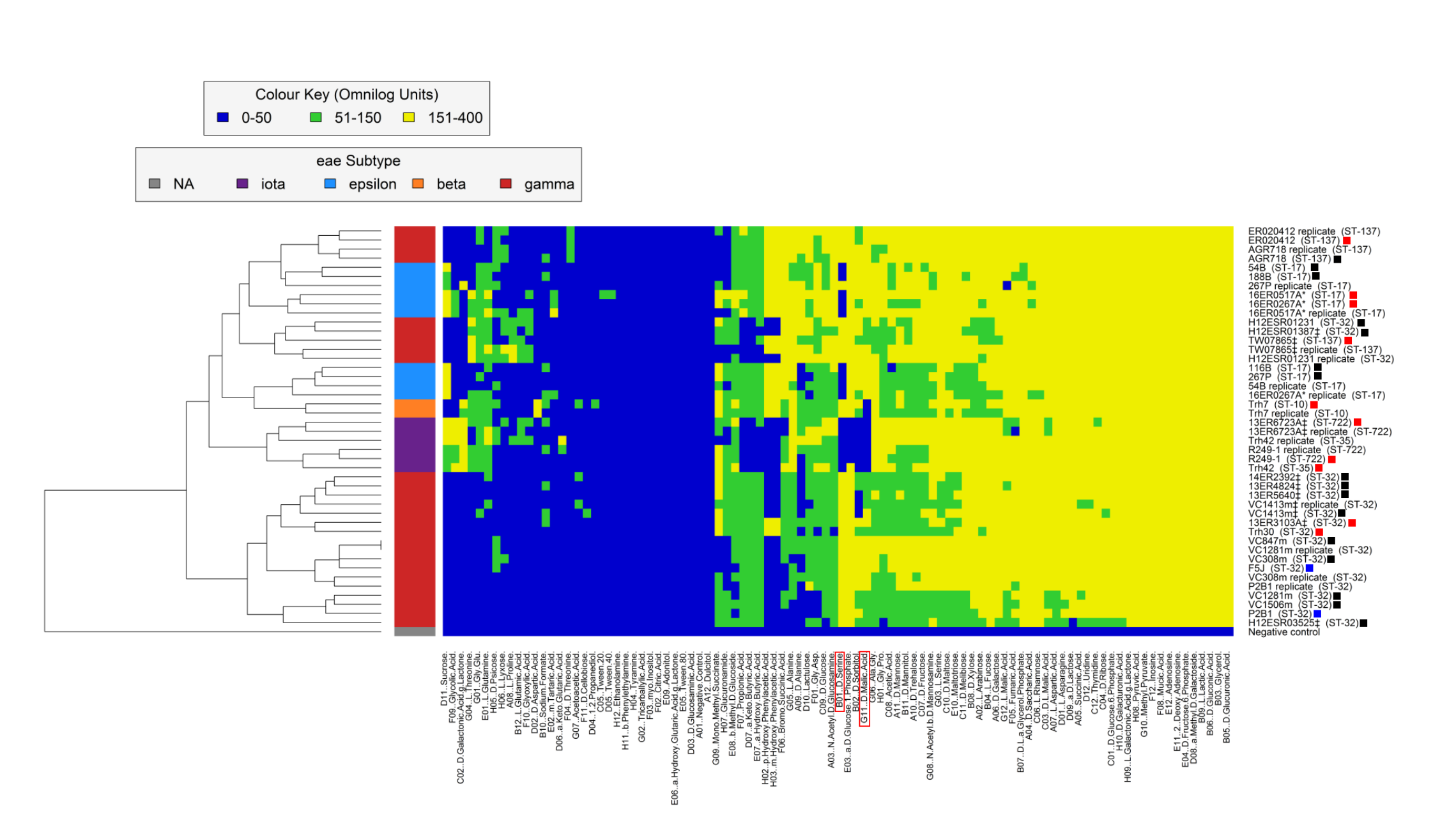

Supplement: S2 Fig — Heat-map of PM1 carbon substrate metabolism over a 24-hour incubation period at 37°C by serogroup O145 strains. The end-point utilisation values (Omnilog Units) were grouped into the following three categories: 0–50 representing no utilisation, 51–150 representing moderate utilisation and 151–400 representing extensive utilisation, as indicated by the colour key. Each strain (n = 28, n = 14 replicates, n = 2 duplicates) is indicated on the right and the 95 carbon substrates listed along at the foot of the figure. Metadata is included for eae subtype, sequence type, source and whether the strains were toxigenic. eae subtype on the left is represented by the colour key, NA is not applicable, sequence type is shown in brackets, isolate source indicated by the colour boxes next to the label name with black, red and blue boxes representing bovine, human and environmental sources, respectively and stx1 positive as * and stx2 positive as ‡. (PNG) [file pone.0235066.s008.png]

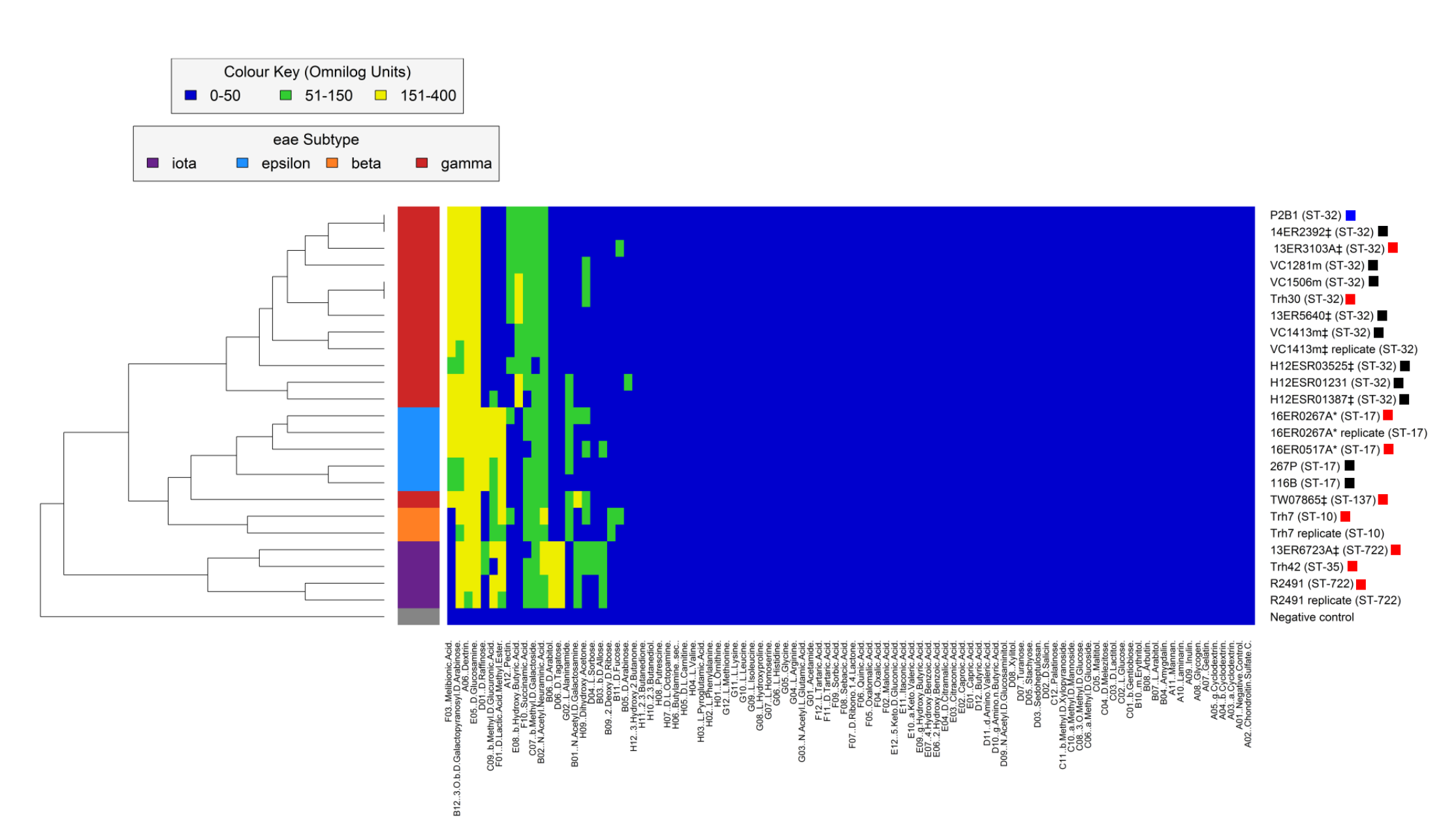

Supplement: S3 Fig — Heat-map of PM2A carbon substrate metabolism over a 24-hour incubation period at 37°C by serogroup O145 strains. The end-point utilisation values (Omnilog Units) were grouped into the following three categories: 0–50 representing no utilisation, 51–150 representing moderate utilisation and 151–400 representing extensive utilisation, as indicated by the colour key. Each strain (n = 20 and n = 4 replicates) is indicated on the right and the 95 carbon substrates listed along at the foot of the figure. Metadata is included for eae subtype, sequence type, source and whether the strains were toxigenic. eae subtype on the left is represented by the colour key, NA is not applicable, sequence type is shown in brackets, isolate source indicated by the colour boxes next to the label name with black, red and blue boxes representing bovine, human and environmental sources, respectively stx1 positive as * and stx2 positive as ‡. (PNG) [file pone.0235066.s009.png]
